# Patient outcomes following AKI and AKD: a population-based cohort study

**DOI:** 10.1186/s12916-022-02428-8

**Published:** 2022-07-20

**Authors:** Huan Wang, Emilie Lambourg, Bruce Guthrie, Daniel R. Morales, Peter T. Donnan, Samira Bell

**Affiliations:** 1 Division of Population Health and Genomics, School of Medicine, University of Dundee, Dundee, DD1 9SY UK; 2 Advanced Care Research Centre, Usher Institute of Population Health Sciences and Informatics, College of Medicine and Veterinary Medicine, University of Edinburgh, Edinburgh, UK; 3 Department of Public Health, University of Southern Denmark, Odense, Denmark; 4 Renal Unit, Ninewells Hospital, Dundee, UK

**Keywords:** Acute kidney injury, Acute kidney disease, Chronic kidney disease, Recovery, Epidemiology

## Abstract

**Background:**

Acute kidney injury (AKI) is common and associated with adverse outcomes as well as important healthcare costs. However, evidence examining the epidemiology of acute kidney disease (AKD)—recently defined as AKI persisting between 7 and 90 days—remains limited. The aims of this study were to establish the rates of early AKI recovery, progression to AKD and non-recovery; examine risk factors associated with non-recovery and investigate the association between recovery timing and adverse outcomes, in a population-based cohort.

**Methods:**

All adult residents of Tayside & Fife, Scotland, UK, with at least one episode of community or hospital-managed AKI using KDIGO creatinine-based definition during the period 1 January 2010 to 31 December 2018 were identified. Logistic regression was used to examine factors associated with non-recovery, and Cox modelling was used to establish associations between AKI recovery timing and risks of mortality and development of de novo CKD.

**Results:**

Over 9 years, 56,906 patients with at least one AKI episode were identified with 18,773 (33%) of these progressing to AKD. Of those progressing to AKD, 5059 (27%) had still not recovered at day 90 post AKI diagnosis. Risk factors for AKD included: increasing AKI severity, pre-existing cancer or chronic heart failure and recent use of loop diuretics. Compared with early AKI recovery, progression to AKD was associated with increased hazard of 1-year mortality and de novo CKD (HR = 1.20, 95% CI 1.13 to 1.26 and HR = 2.21, 95% CI 1.91 to 2.57 respectively).

**Conclusions:**

These findings highlight the importance of early AKI recognition and management to avoid progression to AKD and long-term adverse outcomes.

**Supplementary Information:**

The online version contains supplementary material available at 10.1186/s12916-022-02428-8.

## Background

Globally, 13 million people worldwide are thought to be affected by acute kidney injury (AKI) every year [1]. The incidence is estimated between 7 and 18% amongst hospital in-patients with rates ranging between 30 and 70% in the critically ill [2], making it one of the most common complications following hospital admission. AKI also affects about 400 per 100,000 persons per year in community-based populations with an increasing incidence [3]. It is well established that acute kidney injury (AKI) is associated with adverse outcomes including development or worsening of CKD, [4, 5] kidney failure, cardiovascular events [6, 7], and reduced survival [8]. There is however limited evidence examining post-AKI renal recovery and how short-term recovery affects longer term outcomes. Even though community-acquired may be the most common form of AKI [9], evidence regarding community-acquired/community-managed AKI is sparse. Compared to those managed in-hospital, AKI cases managed in the community could represent a different sample of patients with fewer risk factors, milder cases and better outcomes [10] or conversely a palliative care population. Including these patients allows for a comprehensive depiction of real-word AKI burden and therefore generalizable findings. Over the past 15 years, definitions of both AKI and CKD have been agreed in formal consensus studies, and these definitions are currently applied widely in both research and clinical practice. However, no official definition for AKI recovery currently exists with a lack of consensus on how recovery should be defined [11]. Recently, the term acute kidney disease (AKD) has been proposed by Acute Disease Quality Initiative (ADQI) Workgroup to define an “acute or subacute damage and/or loss of kidney function for a duration of between 7 and 90 days after exposure to an AKI initiating event” [12]. This bridges the gap between AKI and CKD, reflecting increasing recognition that AKI and CKD are interconnected and likely represent a continuum, with patients who have sustained an episode of AKI having an increased risk of either developing de novo CKD or experiencing worsening of underlying CKD [13, 14]. However, important knowledge gaps on the epidemiology including the clinical course of AKD need to be addressed before this terminology can be meaningfully used in clinical practice or research to differentiate early (in the first 7 days) and delayed (between 8 to 90 days) renal recovery after AKI. Furthermore, with the exception of some general key recommendations proposed by KDIGO [15], there are a lack of guidelines targeting AKI and AKD follow-up care.

The aim of this study is to (i) establish the rates of early recovery, progression to AKD and non-recovery following AKI using population based routinely collected healthcare data, (ii) understand which factors are associated with progression to AKD and non-recovery and (iii) explore the relationship between recovery timing and survival as well as development of de novo CKD.

## Methods

### Study population

This was a population-based cohort formed of all adults (aged 18 or above) in Tayside & Fife, Scotland, UK, who had at least two serum creatinine measurements on different days and presented an AKI episode between 1 January 2010 and 31 December 2018. Cohort entry (index date) was defined as the first day of the first AKI diagnosis during the study period.

### Data sources

Data were provided by the Health Informatics Centre (HIC) [16] at the University of Dundee which enables anonymised linkage of health records of all residents of Tayside and Fife, Scotland (population of approximately 800,000 individuals), using the unique Community Health Index (CHI) number, which is used across the whole National Health Service (NHS) healthcare system. The following datasets were linked: creatinine laboratory results (community and hospital), Scottish Morbidity Record of hospital admissions (SMR01), medicines dispensed by community pharmacies, the Scottish Care Initiative-Diabetes Collaboration, National Records of Scotland (NRS) death records and the Scottish Renal Registry.

Linkage to SMR01 data provided information on all hospital admission and discharge dates as well as reasons for admission. Deprivation category was derived from the Scottish Index of Multiple Deprivation [17]. Information on diabetes type and date of diagnosis was obtained from the Scottish Care Information-Diabetes Collaboration [18]. Patients receiving chronic dialysis or with a kidney transplant were identified using the Scottish Renal Registry [19]. Comorbidities were identified at the index date and computed based on past ICD-10 hospitalisation codes using the Quan adaptation [20] of the Deyo Charlson mapping algorithm [21].

### Outcomes

The primary outcomes were AKI recovery/non-recovery, death and progression to chronic KRT (Kidney Replacement Therapy). These were assessed at day 7 and day 90 post AKI diagnosis.

Secondary outcomes included progression to de novo chronic kidney disease and recovery timing—in terms of “days to recovery from the first day of AKI diagnosis”. Secondary outcomes were only assessed in a subset of the cohort: amongst patients without pre-existing chronic kidney disease and amongst patients with hospital-managed AKI respectively. The association between recovery timing and 1 year-mortality as well as 1-year de novo CKD were also explored.

### Definitions

**Fig. 1:**
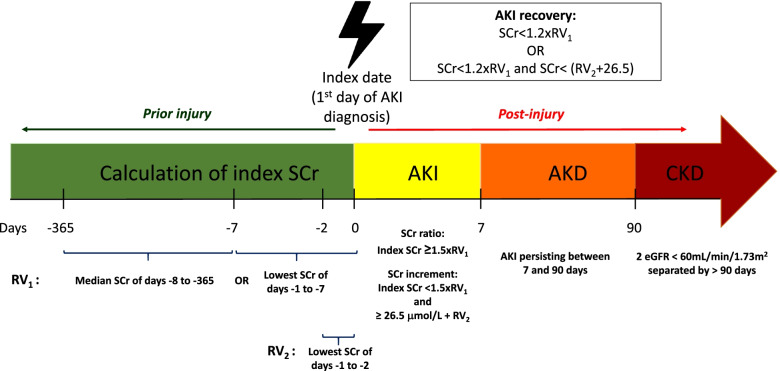
AKI, AKD and CKD definitions

Detailed descriptions of all the concepts defined below are also available in Table 1 and illustrated in Fig. 1.

**Table 1:** Definitions and descriptions for identification of acute kidney injury (AKI) episode

Definition	Description
**Baseline serum creatinine (SCr)**	
Reference value 1 (RV_1_)	Lowest value of: • Median of the SCr levels in the 8 to 365 days prior to the index date • Lowest of the SCr levels in the 1 to 7 days prior to the index date
Reference value 2 (RV_2_)	Lowest value of the SCr levels in the 1 to 2 days (48 h) prior to the index date
**AKI identification**	
By SCr ratio	Index SCr ≥ 1.5 times higher than RV_1_
By SCr increment	Index SCr < 1.5 times higher than RV_1_; but ≥26.5 μmol/L higher than RV_2_
**AKI stage**	
Stage 1	Index SCr/RV_1_ ≥ 1.5 and < 2; or Index SCr – RV_2_ ≥ 26.5 μmol/L
Stage 2	Index SCr/RV_1_ ≥ 2 and < 3
Stage 3	Index SCr/RV_1_ ≥ 3; or Index SCr ≥ 353.6 μmol/L
**AKI category**	
Community-acquired/community-managed AKI	AKI diagnosed in the community and not admitted to hospital within 7 days
Community-acquired/hospital-managed AKI	Either of the following: • AKI developed in the community and admitted to hospital within 7 days • AKI presented in the first 2 days in hospital, i.e. day 0 (admission) + day 1
Hospital-acquired AKI	AKI developed on or after 2 days in hospital, i.e. from day 2 onwards
**Patient status at day 7 and day 90 following the AKI episode**	
Creatinine-recovery	Patient recovered, did not die and did not initiate chronic KRT during the period considered
No creatinine-recovery	Patient did not recover, did not die and did not initiate chronic KRT during the period considered
Death	Patient died during the period considered
KRT	Patient initiated KRT during the period considered
Untested	Patient was not tested, did not die and did not initiate chronic KRT during the period considered

#### Acute kidney injury (AKI)

AKI definition was based on the Kidney Disease: Improving Global Outcomes (KDIGO) creatinine-based criteria [22], using the NHS England AKI e-alert algorithm [23]. The mean creatinine was calculated if there was more than one serum creatinine (SCr) measurement taken on the same day. By linking creatinine measurements to hospital admission data, AKI was further classified into 3 categories: community-acquired/community-managed (CA-CM), community-acquired/hospital-managed (CA-HM) and hospital-acquired (HA). An AKI episode diagnosed in the community was categorised as CA-CM AKI if there was no hospital admission within 7 days post AKI diagnosis. CA-HM AKI was defined as either an AKI episode diagnosed in the community with hospital admission within 7 days post AKI diagnosis or an AKI episode diagnosed on the day (J0) or the next day (J1) following an hospital admission. Finally, the definition of HA AKI was met for patients developing an AKI episode after 2 days in hospital (J2) or later.

#### Acute kidney disease (AKD)

AKD was defined as a loss of kidney function for a duration between 7 and 90 days after exposure to an AKI initiating event, as per the ADQI Workgroup definition [12]. By default, patients tested within the first 7 days post AKI diagnosis who did not meet criteria for recovery at day 7 entered the AKD phase at that point (provided they did not die or initiate chronic KRT before day 7). Their loss of kidney function was described as AKD until either criterion for recovery was met or day 90 after the AKI initiating event, whichever came first.

#### Chronic kidney disease (CKD)

CKD was defined according to the KDIGO definition [24] where eGFR was calculated using the CKD-EPI Creatinine Equation [25] using standardised SCr level.

Therefore, the presence of 2 eGFR records below 60 mL/min/1.73 m2 separated by more than 90 days was used to define CKD. Pre-existing CKD was determined using all SCr measurements strictly prior to the index date (first day of AKI diagnosis) whilst de novo CKD was determined using all SCr measurements sampled strictly after the 90^th^ day following the index date.

Progression to CKD was only investigated in patients who had no pre-existing CKD identified prior to the index date.

#### AKI recovery

Creatinine-based recovery was defined as having a creatinine measurement within 90 days post AKI diagnosis that was either < 1.2 times higher than reference value 1 (RV_1_) (for AKI identified by creatinine ratio) or < 1.2 times higher than RV_1_ and < 26.5 μmol/L higher than reference value 2 (RV_2_) (for AKI identified by creatinine increment) [26]. All SCr measurements within the 90 days post AKI diagnosis were used to search for creatinine-based recovery. The earliest date with a SCr measurement meeting the recovery criteria described above was defined as the date of recovery. In order to avoid misclassification, two additional criteria had to be met to fulfil the definition of creatinine-based recovery: (1) absence of chronic KRT initiation in the 30 days following the date of creatinine recovery and (2) recovery status sustained for at least 3 days (day of creatinine recovery + the two following days—although this could only be applied if tests were available over 3 consecutive days, thereby only avoiding misclassification of detected early relapses as recoveries). Recovery timing was then defined as early if the patient recovered within the first 7 days (day 7 included) or as delayed if criteria for recovery were not met in the first 7 days but were further met during the AKD phase (day 8 to day 90 following AKI diagnosis).

At day 7 and day 90 post AKI diagnosis, patient status was classified into one of the states described in Table 1. Patients who either recovered, died or started chronic KRT during a time period were excluded from the sub-cohort for the next time period (or censored on the date of recovery, death or chronic KRT initiation in survival analyses).

Patients untested within the first 7 days, who did not die or commence chronic KRT during that period, were described but excluded from all statistical analyses as no assumption can be made regarding their recovery status.

#### Chronic KRT

Chronic KRT was defined as either dialysis initiation (haemodialysis or peritoneal dialysis) or kidney transplantation. The date of chronic dialysis initiation or kidney transplantation is recorded in the Scottish Renal Registry for all patients starting chronic KRT in Scotland with 100% coverage.

### Statistical analysis

Characteristics of the study population were summarised by medians and interquartile ranges for continuous measurements (due to non-Normal distributions) and as percentages for categorical factors. Scottish Index of Multiple Deprivation (SIMD) quintiles were summarised as a categorical factor. Age was converted to a categorical variable with approximately similar numbers within each category (less than 65, 65 to 74, 75 to 84, 85+ years old) and youngest patients (< 65 years old) taken as the reference level. Multivariable logistic regression models were implemented to identify risk factors associated with progression to AKD, taking patients with early recovery as the reference level. We then considered patients who entered the AKD phase and determined risk factors associated with non-recovery at day 90 post-AKI diagnosis, using another multivariable logistic model. For both models, we excluded patients who died or initiated chronic KRT, between day 1 and day 7, and between day 8 and day 90 respectively. In a sensitivity analysis, the models were rerun keeping patients who died or initiated chronic KRT during the period considered in the non-recovery group, and risk factors for non-recovery were re-identified. The same candidate risk factors were included in both models: demographic characteristics (age at AKI diagnosis, sex and social deprivation); baseline comorbidities (decreased baseline eGFR, cancer, coronary artery disease, congestive heart failure, diabetes and hypertension) and medications (ACE inhibitors or ARBs, loop diuretics, metformin, NSAIDs, statins) received in the 90 days prior to the index date. Those variables were checked for multicollinearity using a correlation matrix and the variance inflation factor. Frequency of creatinine measurements can provide additional important information that other variables cannot capture and was therefore included in the models as a continuous variable.

Associations between recovery timing and 1-year mortality or de novo CKD were evaluated in the recovery cohort (patients with proven recovery within the 90 days following AKI diagnosis) amongst those who had been tested within the first 7 days, using multivariable Cox proportional hazards (PH) models. People in the recovery cohort were followed up from the recovery date (time 0) until either occurrence of one of the study outcomes (death or de novo CKD) or censored at the last date of data availability (29-05-2019). Development of de novo CKD was assessed using a cause-specific Cox proportional hazard model with all-cause mortality as a competing endpoint. All AKI categories were included when exploring the association between delayed versus early recovery and adverse outcomes. However, the association between days to recovery and adverse outcomes was only investigated in patients with hospital-managed AKI, since the testing frequency (number of SCr measurements divided by number of days from AKI to recovery) was too low in those with community-managed AKI to allow for a precise determination of recovery timing, hence the exclusion of this AKI subgroup from this specific analysis. Days to recovery was included as a continuous variable using P-splines [27] to allow for non-linear effects on the hazard of study outcomes, with reference set as the median recovery time (4 days, HR = 1). Previous work has demonstrated good accuracy of penalised spline smoothing methods to account for nonlinear effects of covariates in Cox models [28]. Selection of the optimal smoothing parameter controlling the penalty applied to the curve was determined on the basis of the Akaike Information Criteria (AIC) [29]. For each individual Cox model, the proportional hazards (PH) assumption was checked using graphical diagnosis based on the scaled Schoenfeld residuals and testing of independence between residuals and time.

All data were analysed using the R statistical programming language (Version 3.6.2, Vienna, Austria) using the following packages: dplyr, data.table, survival, survminer, networkD3, graphics and sjPlot.

## Results

### Description of the cohort

**Fig. 2:**
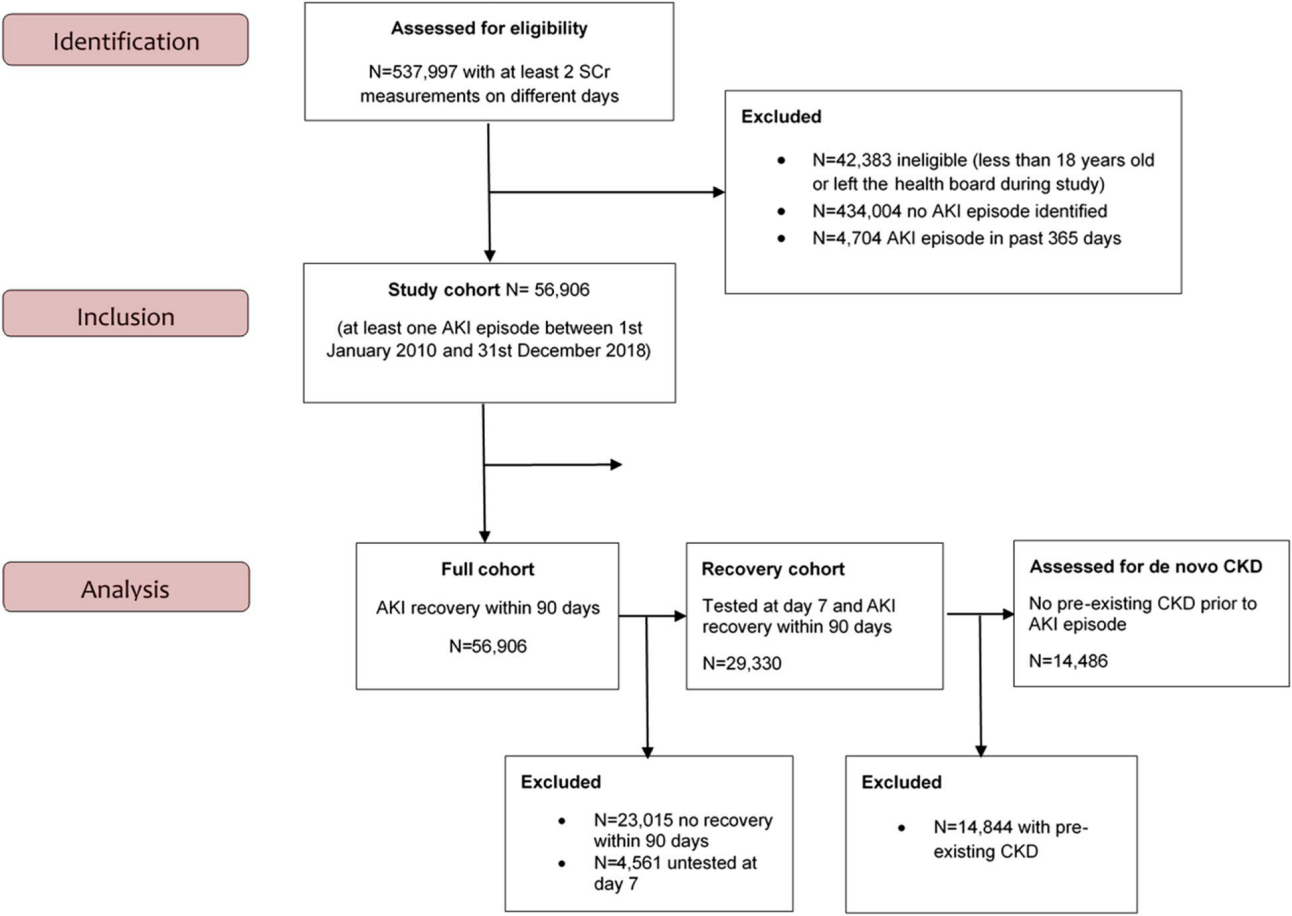
Flow chart of cohort design

The study cohort consisted of 56,906 patients who had at least one AKI episode during the period 1 January 2010 to 31 December 2018 (Fig. 2). They were followed-up for a median time of 2.1 years (IQR: 0.4 to 4.7 years). Of those 56,906 patients, 13,443 (24%) had AKI diagnosed and managed in the community (community-acquired/community-managed), 22,637 (40%) had AKI diagnosed in the community but managed in hospital (community-acquired/hospital-managed), and for 20,826 (36%), AKI was acquired and managed in-hospital (hospital-acquired). Out of all first AKI episodes during the study period, 45,361 (80%) were stage 1 at diagnosis, 7599 (13%) were stage 2, and 3946 (7%) were stage 3. The median age of the cohort was 75 years old (IQR: 63 to 83) with an evenly distributed men/women ratio. Patients with community-acquired/community-managed AKI were younger (median: 69 years old, IQR: 52 to 80), with a larger proportion of women (65%) and fewer comorbidities, compared to those managed in hospital. Patients’ characteristics at baseline stratified by AKI category are summarised in Table 2. Table 3 summarises outcomes at 90 days and 1 year following the AKI episode, stratified by AKI category and AKI severity. At 1-year post-AKI, 18,381 patients (32.3%) had died, with the lowest crude mortality observed amongst those with community-acquired/community-managed AKI (15.8%) followed by community-acquired/hospital-managed AKI (36.4%) and hospital-acquired AKI (38.5%). Additional file 1: Fig. S1 depicts the overall survival following the AKI episode, stratified by AKI category. Mortality was associated with AKI severity, with 1-year survival of 69.8% and 57.3% for those with AKI stage 1 and 3 respectively.

**Table 2:** Patient’s baseline characteristics (%)

	Community-acquired/community-managed AKI (***n*** = 13,443)	Community-acquired/hospital-managed AKI (***n*** = 22,637)	Hospital-acquired AKI (***n*** = 20,826)	All AKI (***n*** = 56,906)
**N SCr tests—day 1 to 7 (median, [IQR])**	0 [0–1]	3 [1–5]	3 [1–5]	2 [0–4]
**N SCr tests—day 1 to 90 (median, [IQR])**	2 [0–4]	6 [3–12]	6 [2–13]	5 [2–10]
**AKI severity at diagnosis**				
Stage 1	11984 (89)	15385 (68)	17992 (86)	45361 (80)
Stage 2	1118 (8)	4412 (19)	2069 (10)	7599 (13)
Stage 3	341 (3)	2840 (13)	765 (4)	3946 (7)
**Age at AKI diagnosis (median, [IQR])**	69 [52–80]	74 [63–83]	78 [67–85]	75 [63–83]
**Sex = male**	4706 (35)	11305 (50)	9875 (47)	25886 (45)
**SIMD quintile**				
1 (most deprived)	2652 (20)	4552 (20)	3690 (18)	10894 (19)
2	2720 (20)	4791 (21)	4018 (19)	11529 (20)
3	2670 (20)	4600 (20)	4111 (20)	11381 (20)
4	3295 (25)	5372 (24)	5605 (27)	14272 (25)
5 (least deprived)	2106 (16)	3322 (15)	3402 (16)	8830 (16)
**AKI identified by**				
SCr ratio	13213 (98)	20907 (92)	15655 (75)	49775 (87)
SCr increment	230 (2)	1730 (8)	5171 (25)	7131 (13)
**Baseline eGFR category**				
≥ 90	5482 (41)	5838 (26)	4515 (22)	15835 (28)
60–89	4489 (33)	8972 (40)	8236 (40)	21697 (38)
45–59	1767 (13)	3808 (17)	3784 (18)	9359 (16)
30–44	1217 (9)	2749 (12)	2949 (14)	6915 (12)
< 30	488 (4)	1270 (6)	1342 (6)	3100 (5)
**Comorbidity**				
Chronic kidney disease (CKD)	4080 (30)	7963 (35)	8588 (41)	20631 (36)
Cancer	3392 (25)	7741 (34)	8064 (39)	19197 (34)
Coronary arterial disease (CAD)	2496 (19)	5521 (24)	5751 (28)	13768 (24)
Congestive heart failure (CHF)	1199 (9)	2425 (11)	3126 (15)	6750 (12)
Diabetes	3465 (26)	6252 (28)	5426 (26)	15143 (27)
Hypertension	3929 (29)	8272 (37)	8576 (41)	20777 (37)
**Medication in prior 90 days**				
ACEi/ARB	5196 (39)	8735 (39)	6892 (33)	20823 (37)
Loop diuretic	3123 (23)	5017 (22)	4324 (21)	12464 (22)
Metformin	1430 (11)	2355 (10)	1720 (8)	5505 (10)
NSAID	1185 (9)	1840 (8)	1213 (6)	4238 (7)
Statin	4549 (34)	8587 (38)	7032 (34)	20168 (35)

**Table 3:** Mortality and chronic KRT initiation at 90 days and 1-year post AKI

	Total number of patients	All-cause mortality		Chronic KRT	
	***N***	90 days	1 year	90 days	1 year
**All AKI**	56,906	12,623 (22.2)	18,381 (32.3)	213 (0.37)	328 (0.58)
**AKI subgroup**					
Community-acquired/community-managed AKI	13,443	992 (7.4)	2122 (15.8)	48 (0.36)	99 (0.74)
Community-acquired/hospital-managed AKI	22,637	5946 (26.3)	8247 (36.4)	96 (0.42)	135 (0.60)
Hospital-acquired AKI	20,826	5685 (27.3)	8012 (38.5)	69 (0.33)	94 (0.45)
**AKI severity**					
Stage 1	45,361	9036 (19.9)	13,694 (30.2)	29 (0.064)	81 (0.18)
Stage 2	7599	2306 (30.3)	3003 (39.5)	8 (0.10)	18 (0.24)
Stage 3	3946	1281 (32.5)	1684 (42.7)	176 (4.5)	229 (5.8)
**All AKD**	18,773	3695 (19.7)	5927 (31.6)	135 (0.72)	205 (1.1)

From the 56,906 patients in the cohort, only 535 (0.94%) commenced chronic KRT after the AKI episode. This was strongly associated with AKI severity, with 6.9% of patients with AKI stage 3 further initiating chronic KRT.

Additional file 1: Table S1 summarises recovery status at 7 and 90 days post AKI, stratified by AKI category and AKI stage at diagnosis. During the first 7 days post AKI diagnosis, 20,041 (35.2%) out of 56,906 recovered, 18,773 (33.0%) were tested but had not recovered, 13,154 (23.1%) were not tested, 4892 (8.6%) died and 46 (0.08%) initiated chronic KRT. Proven recovery rate was highest in people with community-acquired/hospital-managed AKI (47.0%), followed by hospital-acquired (38.4%), and was only 10.4% in people with community-acquired/community-managed AKI. However, a large proportion (66.7%) of patients with community-acquired/community-managed AKI were not tested in the first 7 days, which was not the case amongst those with community-acquired/hospital-managed AKI (8.7% untested) or hospital-acquired AKI (10.7% untested). In a sensitivity analysis, we compared the characteristics of patients with community-acquired/community-managed AKI who were tested versus untested within the first 7 days post-AKI. This analysis, which only included those who had survived and not initiated chronic KRT at day 7, showed that untested patients tended to be younger (median age: 68 vs 72 years old), with fewer comorbidities, a higher baseline eGFR (eGFR> 90 in 47% vs 29%) and milder AKI (stage 1: 93% vs 83%) compared with tested patients (Additional file 1: Table S2). Compared with community-managed AKI, those with hospital-managed AKI (community- and hospital-acquired) were also more often tested within 90 days post-AKI (median number of SCr tests: 6 versus 2).

At day 8, 18,773 (33%) patients from the initial cohort entered the AKD cohort. Of these, 7698 (41%) had a delayed recovery, with a similar proportion in the different AKI categories, whilst 5059 (27%) had still not recovered at day 90. A total of 3695 (19.7%) patients with AKD died between day 8 and day 90, with a higher proportion amongst those who were managed in hospital (21.8% for community-acquired/hospital-managed AKI and 21.4% for hospital-acquired AKI versus 8.7% for community-acquired/community-managed AKI). Of note, 11.7% of those who had been tested but had not recovered at day 7 were not retested between day 8 and day 90 whilst 36.2% of those who had not been tested within the first 7 days had still not been tested at day 90 (Additional file 1: Table S3).

**Fig. 3:**
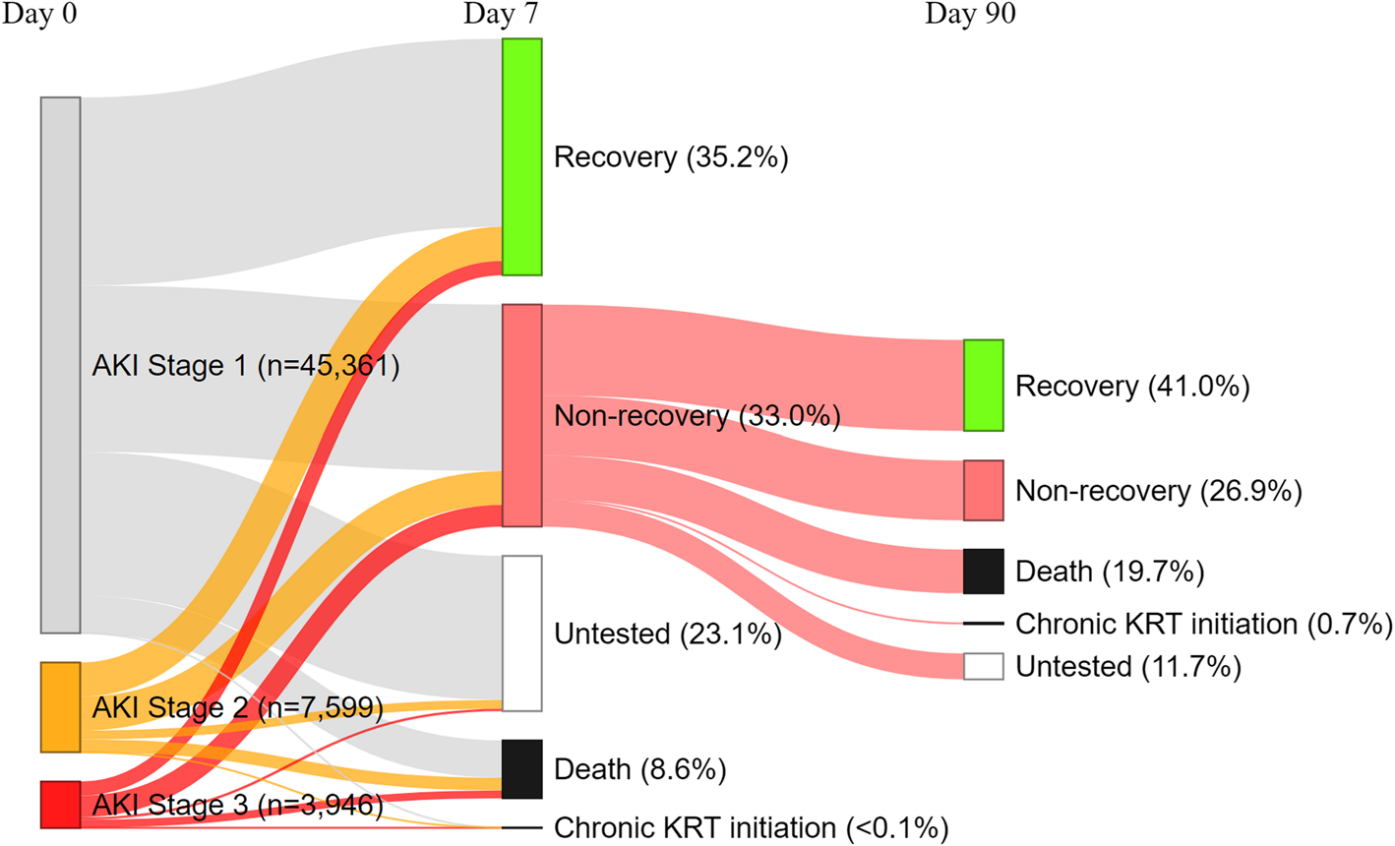
Sankey diagrams showing patient status at 7 and 90 days post-AKI diagnosis (all AKI categories)

**Fig. 4:**
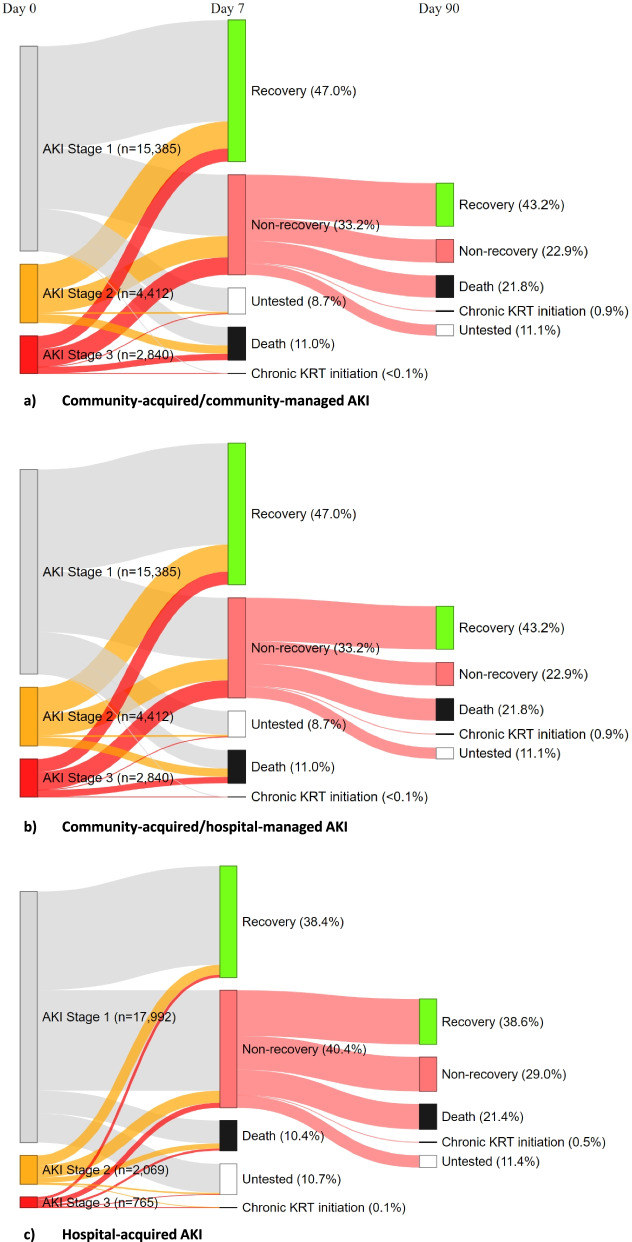
**a–c** Sankey diagrams showing patient status at 7 and 90 days post-AKI diagnosis, by AKI category

Day 7 status for the whole cohort, as well as day 90 status for those who entered the AKD phase, can be visualised in the Sankey diagrams, with and without stratification by AKI categories (Figs. 3 and 4a,b,c respectively).

### Factors associated with progression to AKD and non-recovery

Risk factors associated with progression to AKD amongst tested individuals are summarised in Table 4 and Additional file 1: Figure S2 for the main analysis, in Additional file 1: Table S4 and Figure S3 for the sensitivity analysis (in which patients who died or initiated chronic KRT within the first 7 days were not excluded but rather considered as having not recovered during that period). More severe AKI at diagnosis (stages 2 and 3), a history of cancer diagnosis, a history of congestive heart failure and recent exposure to loop diuretics or metformin were significantly associated with progression to AKD. Conversely, prior exposure to ACE/ARB was associated with early AKI recovery (adjusted OR = 0.85, 95% CI 0.81 to 0.89, *p* < 0.001). The adjusted odds of progressing to AKD were 2.3 times higher (95% CI 2.2 to 2.5) in those with community-acquired/community-managed AKI than in those with community-acquired/hospital-managed AKI. An older age was negatively associated with progression to AKD, however this trend disappeared in the sensitivity analysis.

**Table 4:** Association between individual predictors and progressing to AKD, excluding patients who died or initiated chronic KRT within the first 7 days

		Unadjusted		Adjusted	
		Odds ratio (95% CI)	***p***-value	Odds ratio (95% CI)	***p***-value
	*N*	38,814		38,814	
**Progressed to AKD,*N*(%)**	18,773 (33)			18,773 (33)	
**Age at AKI diagnosis**					
< 65 (ref)	9665	–	–	–	–
65–74	8491	0.88 (0.83–0.93)	< 0.001	0.94 (0.88–1.01)	0.09
75–84	11,781	0.75 (0.71–0.80)	< 0.001	0.85 (0.80–0.91)	< 0.001
85+	8877	0.68 (0.65–0.72)	< 0.001	0.77 (0.71–0.83)	< 0.001
**Sex = male**	18,529	0.96 (0.92–1.00)	0.05	1.05 (1.01–1.10)	0.02
**SIMD quintile**					
1 (ref)	7214	–	–	–	–
2	7720	0.98 (0.92–1.04)	0.5	1.00 (0.93–1.07)	0.9
3	7764	0.94 (0.88–1.00)	0.06	0.97 (0.91–1.04)	0.3
4	10,033	0.93 (0.87 to 0.99)	0.02	0.95 (0.89–1.02)	0.1
5	6083	0.98 (0.92–1.05)	0.6	1.02 (0.95–1.10)	0.6
**AKI category**					
CA-HM (ref)	18,172	–	–	–	–
CA-CM	4223	2.86 (2.66–3.07)	< 0.001	2.34 (2.17–2.53)	< 0.001
HA	16,419	1.49 (1.43–1.55)	< 0.001	1.91 (1.82–2.00)	< 0.001
**AKI identified by**					
SCr ratio (ref)	33,032	–	–	–	–
SCr increment	5782	0.47 (0.44–0.50)	< 0.001	0.45 (0.42–0.48)	< 0.001
**AKI severity at diagnosis**					
Stage 1 (ref)	30,006	–	–	–	–
Stage 2	5773	1.13 (1.06–1.19)	< 0.001	1.23 (1.15–1.30)	< 0.001
Stage 3	3035	1.72 (1.59–1.86)	< 0.001	2.40 (2.21–2.61)	< 0.001
**Number of tests**	Median = 3 [IQR: 2–5]	0.88 (0.87–0.89)	< 0.001	0.89 (0.88–0.90)	< 0.001
**Baseline eGFR category**					
≥ 90 (ref)	8994	–	–	–	–
60–89	15,226	0.79 (0.75–0.83)	< 0.001	0.92 (0.87–0.98)	0.01
45–59	6967	0.64 (0.60–0.68)	< 0.001	0.82 (0.76–0.89)	< 0.001
30–44	5282	0.61 (0.57–0.65)	< 0.001	0.84 (0.77–0.91)	< 0.001
< 30	2345	0.68 (0.63–0.75)	< 0.001	0.96 (0.86–1.07)	0.4
**Comorbidity**					
Cancer	13,441	1.13 (1.09–1.18)	< 0.001	1.22 (1.17–1.28)	< 0.001
CAD	9799	0.90 (0.86–0.94)	< 0.001	0.96 (0.91–1.02)	0.2
CHF	4847	1.14 (1.07–1.21)	< 0.001	1.33 (1.24–1.42)	< 0.001
Diabetes	10,799	1.01 (0.96–1.05)	0.8	1.02 (0.96–1.08)	0.5
Hypertension	14,908	0.90 (0.86–0.93)	< 0.001	0.98 (0.93–1.02)	0.3
**Medication in prior 90 days**					
ACEi/ARB	14,826	0.84 (0.81–0.88)	< 0.001	0.85 (0.81–0.89)	< 0.001
Loop diuretic	8610	1.02 (0.97–1.07)	0.4	1.12 (1.06–1.18)	< 0.001
Metformin	3912	1.15 (1.07–1.23)	< 0.001	1.23 (1.14–1.34)	< 0.001
NSAID	2717	0.96 (0.89–1.04)	0.3	0.87 (0.80–0.94)	< 0.001
Statin	14,340	0.88 (0.84–0.92)	< 0.001	0.95 (0.91–1.00)	0.06

The results also showed that a higher number of SCr tests performed over the first 7 days was associated with early AKI recovery (OR = 0.89 for one supplementary test, 95% CI 0.88 to 0.90, *p* < 0.001). Risk factors associated with non-recovery at day 90 are summarised in Table 5 and Additional file 1: Figure S4 for the main analysis, in Additional file 1: Table S5 and Figure S5 for the sensitivity analysis (in which patients who died or initiated chronic KRT between day 8 and day 90 were not excluded but rather considered as having not recovered during that period). Later AKI stages, hospital-acquired/hospital-managed AKI, community-acquired/community-managed AKI, a history of cancer or chronic heart failure increased the odds for non-recovery in the main and sensitivity analyses. Prior recent exposure to ACE/ARB was also consistently associated with proven recovery at day 90 (aOR = 0.84, 95% CI 0.77 to 0.92 in main analysis, aOR = 0.76, 95% CI 0.70 to 0.81 in the sensitivity analysis). Lower baseline eGFR values were associated with recovery during the AKD phase.

**Table 5:** Association between individual predictors and non-recovery at day 90 in patients who entered the AKD phase, excluding patients who died or initiated chronic KRT between day 8 and day 90

		Unadjusted		Adjusted	
		OR (95% CI)	***p***-value	OR (95% CI)	***p***-value
	*N*	12,757		12,757	
**No recovery at day 90,*N*(%)**		5059 (39.7%)		5059 (39.7%)	
**Age at AKI diagnosis**					
< 65 (ref)	3592	–	–	–	–
65–74	3001	0.75 (0.68–0.82)	< 0.001	0.97 (0.87–1.08)	0.6
75–84	3781	0.68 (0.62–0.74)	< 0.001	1.02 (0.90–1.15)	0.8
85+	2383	0.58 (0.52–0.65)	< 0.001	0.89 (0.78–1.02)	0.1
**Sex = male**	6110	0.83 (0.78–0.90)	< 0.001	0.87 (0.81–0.94)	< 0.001
**SIMD quintile**					
1 (ref)	2382	–	–	–	–
2	2515	1.03 (0.92–1.16)	0.6	1.06 (0.94–1.19)	0.4
3	2504	1.01 (0.90–1.13)	0.9	1.04 (0.92–1.17)	0.5
4	3293	1.04 (0.94–1.16)	0.4	1.07 (0.96–1.20)	0.2
5	2063	1.08 (0.96–1.22)	0.2	1.12 (0.99–1.27)	0.08
**AKI category**					
CA-HM (ref)	4976	–	–	–	–
CA-CM	2081	1.40 (1.26–1.55)	< 0.001	1.40 (1.25–1.57)	< 0.001
HA	5700	1.41 (1.31–1.53)	< 0.001	1.53 (1.40–1.67)	< 0.001
**AKI identified by**					
SCr ratio (ref)	11,456	–	–	–	–
SCr increment	1301	0.58 (0.51–0.66)	< 0.001	0.71 (0.62–0.81)	< 0.001
**AKI severity at diagnosis**					
Stage 1 (ref)	9524	–	–	–	–
Stage 2	1948	1.05 (0.95–1.15)	0.4	1.11 (1.00–1.24)	0.05
Stage 3	1285	1.11 (0.98–1.25)	0.09	1.43 (1.26–1.63)	< 0.001
**Number of tests**	Median = 5 [IQR: 2–11]	0.97 (0.97–0.97)	< 0.001	0.97 (0.96–0.97)	< 0.001
**Baseline eGFR category**					
≥ 90 (ref)	3241	–	–	–	–
60–89	5149	0.66 (0.60–0.72)	< 0.001	0.69 (0.62–0.76)	< 0.001
45–59	2159	0.47 (0.42–0.53)	< 0.001	0.51 (0.45–0.59)	< 0.001
30–44	1547	0.32 (0.28–0.36)	< 0.001	0.36 (0.31–0.42)	< 0.001
< 30	661	0.33 (0.27–0.40)	< 0.001	0.38 (0.31–0.48)	< 0.001
**Comorbidity**					
Cancer	4349	1.07 (0.99–1.15)	0.08	1.14 (1.05–1.23)	0.001
CAD	3091	0.71 (0.66–0.78)	< 0.001	0.86 (0.78–0.95)	0.002
CHF	1638	0.82 (0.74–0.92)	0.06	1.20 (1.06–1.36)	0.004
Diabetes	3723	0.76 (0.70–0.82)	< 0.001	0.91 (0.82–1.01)	0.07
Hypertension	4802	0.80 (0.75–0.87)	0.003	1.04 (0.96–1.13)	0.3
**Medication in prior 90 days**					
ACEi/ARB	5027	0.70 (0.65–0.76)	< 0.001	0.84 (0.77–0.92)	< 0.001
Loop diuretic	2790	0.66 (0.61–0.72)	< 0.001	0.86 (0.78–0.95)	0.003
Metformin	1542	0.80 (0.71–0.89)	< 0.001	0.98 (0.85–1.14)	0.8
NSAID	880	0.87 (0.75–1.00)	0.05	0.74 (0.64–0.86)	<0.001
Statin	4801	0.74 (0.69–0.79)	<0.001	0.96 (0.87–1.04)	0.3

Age was linearly associated with increased odds of non-recovery at day 90 in the sensitivity analysis only. However, community-acquired/community-managed AKI was no longer a risk factor for non-recovery at day 90 in the sensitivity analysis (aOR = 0.99, 95% CI 0.90 to 1.09).

No multicollinearity was detected between the different predictors investigated, with all correlation coefficients below 60% (Additional file 1: Figure S6).

### Timing of recovery and long-term outcomes

Tested people with either early or delayed proven recovery formed a recovery cohort (*n* = 29,330) with 14,486 individuals free of pre-existing CKD. Of those, 2805 (19.4%) subsequently developed de novo CKD with similar proportions across the different AKI categories (19.3%, 257/1334 of those with community-acquired/community-managed AKI, 19.4% 1534/7890 of those with community-acquired/hospital-managed AKI, and 19.3% 1014/5262 of those with hospital-acquired AKI).

**Fig. 5:**
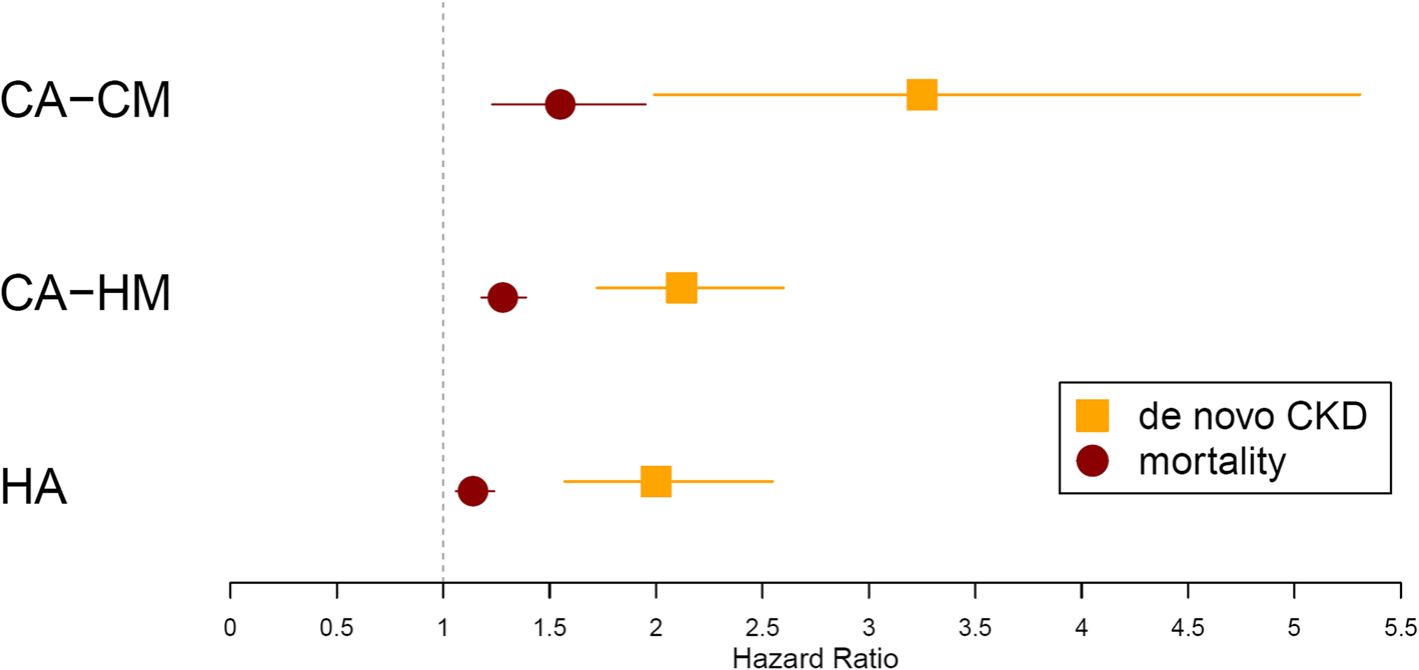
Forest plot displaying risks of 1-year mortality and de novo CKD associated with progression to AKD compared to early recovery

Compared to early recovery, delayed recovery was significantly associated with higher risk of death (HR = 1.20, 95% CI 1.13 to 1.26) and de novo CKD (HR = 2.21, 95% CI 1.91 to 2.57) in the subsequent year following the AKI episode (Additional file 1: Table S6, and Fig. 5). This trend was observed in all AKI categories, but the risk was highest in those with community-acquired/community-managed AKI (HR = 1.55, 95 % CI 1.23 to 1.95 for 1-year mortality and HR = 3.25, 95% CI 1.99 to 5.31 for 1-year risk of de novo CKD). Cox analyses showed that the association between delayed recovery and adverse outcomes was time-varying, with the strongest risks observed over the year following the AKI episode and subsequent wearing off, and no significant association after 2 years.

**Fig. 6:**
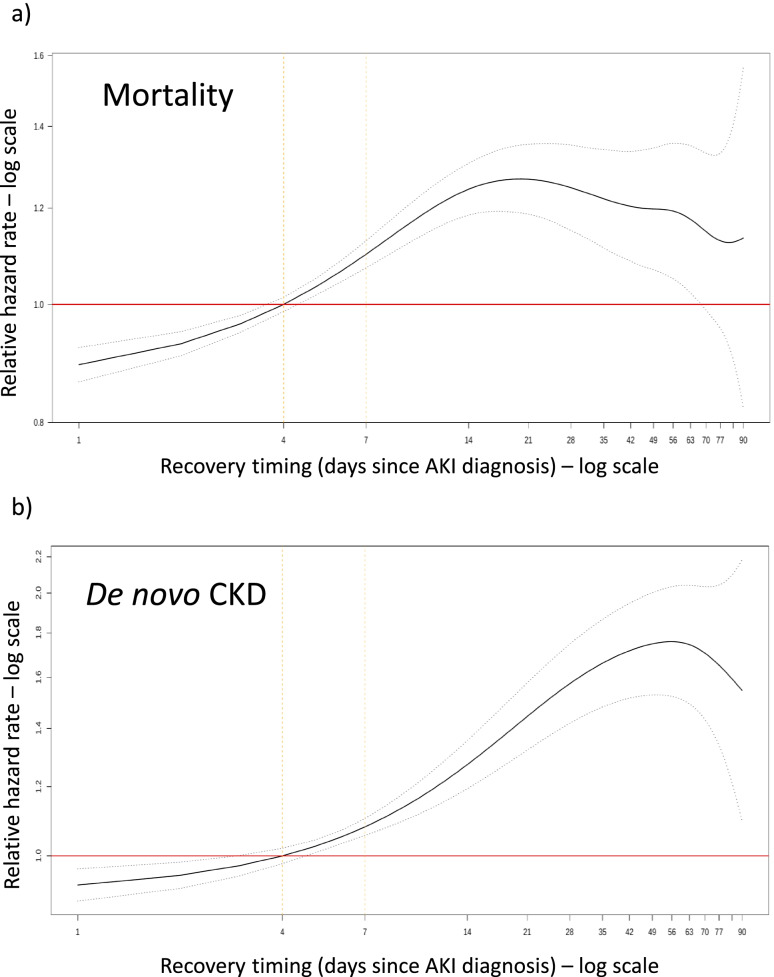
**a**, **b** Association between recovery timing and 1-year relative hazard of death (**a**) and development of de novo CKD (**b**) in patients with hospital-managed AKI

Figure 6 shows the association between all values of recovery timing comprised within 1 and 90 days and relative rates of 1-year mortality (a) as well as development of de novo CKD (b) in patients with hospital-managed AKI (including community-acquired/hospital-managed and hospital-acquired AKI) tested within the first 7 days. Since we would not be able to derive an accurate recovery timing for patients with community-acquired/community-managed AKI (due to the lack of repeat testing), they were excluded from this analysis as well as patients from any AKI category that were untested within the first 7 days post AKI diagnosis. The relative hazard for 1-year mortality increased with recovery timing in a nonlinear fashion, with a sharp initial rise over the first 14 days followed by a plateau. The risk of developing de novo CKD increased more progressively and linearly with recovery timing over the first month following the AKI episode. Beyond this period the risk then stabilised or may even decline.

## Discussion

In this large comprehensive population-based cohort study, there were 56,906 patients with community or hospital-acquired AKI, with a median follow-up of 2.1 years. Overall, 35% of the initial cohort had proven creatinine-recovery at day 7 and 49% at day 90 post AKI diagnosis. Risk factors for progression to AKD included AKI severity, pre-existing cancer or chronic heart failure, recent use of loop diuretics, community-managed AKI as well as hospital-acquired AKI. Of note, being exposed to ACE/ARB was consistently associated with AKI recovery at both day 7 and day 90 (adjusted OR: 0.85, 95% CI 0.81-0.89 and 0.86, 95% CI 0.78–0.95 respectively). Compared with early AKI recovery, progression to AKD was associated with increased risks of 1-year mortality and de novo CKD (HR = 1.20, 95% CI 1.13 to 1.26 and HR = 2.21, 95% CI 1.91 to 2.57 respectively). The first 14 days following an AKI episode were identified as a critical window where each additional day was associated with a rapid increase in risk for adverse outcomes.

It is concerning that in our cohort, a remarkably high proportion of patients with community-acquired/community-managed AKI (67%) were untested at day 7. Amongst those, 36% remained untested at day 90. Patients untested within the first 7 days appeared to be younger, with a higher baseline eGFR and milder AKI which may explain the lack of repeat testing in this fitter population. Furthermore, it is worth noting that in this area of Scotland, repeat testing within 7 days post AKI diagnosis only became more common after the introduction of the National Health Service England Acute Kidney Injury electronic alert algorithm in 2015 [30]. Therefore, a major part of our data captures the practices in place prior to the introduction of this system. Although testing is not always appropriate, for example in palliative care settings or in the context of particularly frail patients, these findings raise questions regarding the management of AKI in the community setting. Moreover, we showed that AKI severity, history of cancer, chronic heart failure and receiving loop diuretics were consistent risk factors for progression to AKD. In line with our result, a previous study aiming to predict recovery following dialysis-requiring AKI showed that patients who recovered were less likely to have a history of heart failure [31]. The same study also identified younger age as a predictor for recovery. Our sensitivity analysis showed that an older age was associated with an increased risk of early death following the AKI episode but not with non-recovery. In both unadjusted and adjusted analyses, we consistently found that prior use of ACE/ARB was significantly associated with AKI recovery at both day 7 and day 90. The use of ACE/ARB in the context of AKI is widely debated. The KDIGO recommendation is to stop potentially nephrotoxic drug (including ACE/ARB in this category) during AKI. However, emerging evidence suggests that ACE/ARB should not be considered as nephrotoxic [32] and could even be associated with improved AKI recovery and reduced subsequent mortality [33–35]. The association between NSAIDs exposure and non-recovery was either non-significant or protective at both day 7 and day 90. NSAIDs-related AKI remain rare events and this observed association may be related to some residual confounding by indication where physicians avoid prescribing NSAIDs to frailer patients they perceive to be at higher risk of NSAIDs-related adverse outcomes such as AKI [36]. Another hypothesis for this finding is the usually rapid renal recovery of NSAIDs-induced AKI (typically within 72 to 96 h provided diagnosis is made early and NSAIDs are promptly discontinued) [37]. Surprisingly, our models suggested an association between lower baseline eGFR values and AKI recovery. This may be because those with lower baseline eGFR values will have greater fluctuations in serum creatinine related to volume status. Recent metformin use appeared as a risk factor for AKD but then as a strong protective factor in our sensitivity analysis when considering non-recovery at day 90. The latter is consistent with previous work reporting improved short-term survival following incident AKI in those exposed to metformin [38, 39].

The association between AKI, AKD and CKD is complex and mortality as well as progression to CKD after an AKI episode have been documented in many studies [8]. Our results showed that compared with early AKI recovery, progression to AKD was associated with both 1-year mortality and development of de novo CKD. This is consistent with previously published data conducted amongst patients admitted for cardiovascular reasons, which demonstrated that AKD was associated with both short- (90 days) [40] and long-term (5 years) [41] risk of death and adverse renal events. However, in a cohort of patient admitted for sepsis-associated AKI, individuals with early AKI reversal had similar mortality rates as those developing AKD [42].

Similarly, we found that risk of death and de novo CKD increased progressively with recovery timing. This is in line with previous work conducted in a cohort of adult US veterans, suggesting that recovery timing may act as an independent predictor for future loss of kidney function [43]. Bhatraju et al. found that recovering within the first 72 h immediately following the AKI episode may be crucial to avoid major adverse kidney events [44]. Compared to a rapid reversal (within 48 h), persistent AKI was also significantly associated with a higher 1-year mortality rate [45]. Recovery timing therefore appears to be a major factor in the context of AKI recovery, which adds important prognostic information regarding adverse long-term outcomes following an AKI episode. In this study, modelling of precise recovery timing showed that the first 2 to 3 weeks following an AKI episode represent a critical window where risk for adverse outcomes increase most rapidly and where interventions are therefore most likely to reduce risk of progression to CKD or early mortality. Mechanisms potentially explaining the association between longer recovery timing and worse outcomes include persistent inflammation, prolonged renin-angiotensin system activation with long-term hypertension even after recovery and repeated cellular injury due to local ischemia leading to kidney damage such as tubular or glomerular injury [46].

Our study has several strengths. These include the comprehensive nature of the unselected population-based cohort covering a large geographical population of Scotland (about 790,000 individuals), the large number of AKI episodes recorded over a 9-year period and the robust methodology accounting for major confounders, with sensitivity analyses ensuring the consistency of findings. The inclusion of community-managed AKI brings new insights regarding level of care and risks associated with treatment outside hospitals, for which data are currently lacking. Finally, our strict definition of sustained recovery reduces misclassification of relapse as recovery. This work helps fill important knowledge gaps in the current understanding of renal recovery after AKI but also comes with a number of limitations. Firstly, this study was conducted in a specific geographic area of Scotland and may not be generalizable to other AKI cohorts worldwide. However, it remains an unselected population-based cohort whose characteristics are similar to that of previous studies and therefore generalizable to other high-income countries. It should be noted that a large proportion (67%) of patients with community-managed AKI were untested during the first 7 days post AKI diagnosis; hence, all conclusions made on this subgroup were based on the subset of patients who had available follow-up SCr data, with subsequent risks of ascertainment and selection bias. However, our sensitivity analysis showed that patients with community-acquired/community-managed AKI tested within the first 7 days have very similar characteristics to that of patients with hospital-acquired AKI, making comparisons relevant. It should be noted that in the absence of any accepted definition [11], we chose the definition of AKI recovery (< 1.2 times higher than baseline SCr) as per previous work [26] but other studies in the field may have used different thresholds, making between-study comparisons less straightforward. Furthermore, we chose to focus on AKD occurring after an AKI event and do not examine AKD occurring without a preceding AKI episode [47]. Another limitation of this study is the lack of data availability regarding the use of temporary dialysis for AKI management, with subsequent risk of AKI recovery misclassification in a small proportion of hospital-managed AKI episodes. Finally, due to the observational nature of this study, risk of residual confounding remains, despite our efforts to control for all important variables.

## Conclusions

Our data demonstrates that AKD is common in patients with AKI and associated with deleterious outcomes such as early mortality or de novo CKD, especially when AKI management takes place outside hospitals. Patients with community-managed AKI should be more widely tested within the first 7 days post AKI diagnosis to ensure optimal management. As risks for adverse outcomes increase sharply during the immediate period (2 to 3 weeks) following AKI diagnosis, this work stresses the importance of early AKI recovery to avoid long-term consequences. Patients with cancer, chronic heart failure and those exposed to diuretics may be at particularly high risk of progression to AKD and non-recovery, therefore deserving extra attention. Although more evidence is needed to guide clinical practice, our results suggested that ACE/ARB may have a protective effect in a context of AKI, with improved recovery amongst recently exposed individuals. Increased awareness and strategies for the management of patients with AKD are needed to maximise early recovery and minimise AKI-related harms.

## Supplementary Information

**Additional file 1: Figure S1**: Survival curve stratified by AKI category. **Table S1**: Recovery status at 7- and 90-days post-AKI diagnosis. **Table S2**: Characteristics of tested versus untested patients with CA-CM AKI. **Table S3**: 90-day status of patients untested within the first 7 days post AKI diagnosis. **Figure S2**: Association between individual predictors and progression to AKD (main analysis). **Table S4** and **Figure S3**: Association between individual predictors and non-recovery at day 7 (sensitivity analysis). **Figure S4**: Association between individual predictors and non-recovery at day 90 in patients who entered the AKD phase (main analysis). **Table S5** and **Figure S5**: Association between individual predictors and non-recovery at day 90 in patients who entered the AKD phase (sensitivity analysis). **Figure S6**: Correlation matrix for pairs of candidate risk factors. **Table S6**: Association between progression to AKD and subsequent risk of death or development of de novo CKD.

## Data Availability

The data controller of the data analysed is NHS Tayside. Patient level data are available subject to standard information governance requirements for use of anonymised, unconsented NHS data https://www.dundee.ac.uk/hic/.

## Abbreviations

ACE/ARB: Angiotensin-converting enzyme inhibitors and angiotensin receptor blockers
ADQI: Acute Disease Quality Initiative
AKD: Acute kidney disease
AKI: Acute kidney injury
CA-CM AKI: Community-acquired/community-managed AKI
CA-HM AKI: Community-acquired/hospital-managed AKI
CKD: Chronic kidney disease
eGFR: Estimates glomerular filtration rate
HA AKI: Hospital-acquired/hospital-managed AKI
HIC: Health Informatics Centre
HR: Hazard ratio
ICD-10: International Classification of Diseases – version 10
IQR: Interquartile range
KDIGO: Kidney Disease Improving Global Outcomes
KRT: Kidney replacement therapy
KTR: Kidney transplant recipients
NHS: National Health Services
NRS: National Record of Scotland
NSAIDs: Nonsteroidal anti-inflammatory drugs
OR: Odds ratio
RV: Reference value
SCr: Serum creatinine
SIMD: Scottish Index of Multiple Deprivation
SMR01: Scottish Morbidity Record of hospital admissions
UK: United Kingdom
US: United States
