# Supplementary material for: Patient outcomes following AKI and AKD: a population-based cohort study
Source: BMC Med. 2022 Jul 20;20:229. doi: 10.1186/s12916-022-02428-8 (PMC9297625; doi:10.1186/s12916-022-02428-8)
Supplement: Supplementary file 1 — Additional file 1: Figure S1: Survival curve stratified by AKI category. Table S1: Recovery status at 7- and 90-days post-AKI diagnosis. Table S2: Characteristics of tested versus untested patients with CA-CM AKI. Table S3: 90-day status of patients untested within the first 7 days post AKI diagnosis. Figure S2: Association between individual predictors and progression to AKD (main analysis). Table S4 and Figure S3: Association between individual predictors and non-recovery at day 7 (sensitivity analysis). Figure S4: Association between individual predictors and non-recovery at day 90 in patients who entered the AKD phase (main analysis). Table S5 and Figure S5: Association between individual predictors and non-recovery at day 90 in patients who entered the AKD phase (sensitivity analysis). Figure S6: Correlation matrix for pairs of candidate risk factors. Table S6: Association between progression to AKD and subsequent risk of death or development of de novo CKD. [file 12916_2022_2428_MOESM1_ESM.docx]

**ADDITIONAL FILES**

**Table of contents**

**Additional File 1: Figure S1:** Kaplan-Meier survival curve comparing post-AKI survival according to AKI category.

**Additional File 2**: **Table S1**: Patients’ recovery status at 7- and 90-days post-AKI (%)

**Additional File 3:** **Table S2:** Characteristics of patients with community-acquired/community-managed AKI tested versus untested within the first seven days post AKI diagnosis.

**Additional File 4:** **Table S3:** 90-day status of patients untested within the first 7 days post AKI diagnosis.

**Additional File 5:** **Figure S2**: Forest plot displaying the association between individual predictors and progression to AKD, excluding patients who died or initiated chronic KRT within the first 7 days.

**Additional File 6: Table S4**: Results of univariable and multivariable logistic regression assessing the association between individual predictors and non-recovery at day 7, including patients who died or initiated chronic KRT within the first 7 days in the non-recovery group.

**Additional File 6: Figure S3**: Forest plot displaying the association between individual predictors and non-recovery at day 7, including patients who died or initiated chronic KRT within the first 7 days in the non-recovery group.

**Additional File 7:** **Figure S4**: Forest plot displaying the association between individual predictors and non-recovery at day 90 in patients who entered the AKD phase, excluding patients who died or initiated chronic KRT between day 8 and day 90.

**Additional Files 8: Table S5**: Results of univariable and multivariable logistic regression assessing the association between individual predictors and non-recovery at day 90 in patients who entered the AKD phase, including patients who died or initiated chronic KRT between day 8 and day 90 in the non-recovery group.

**Additional File 8:** **Figure S5**: Forest plot the association between individual predictors and non-recovery at day 90 in patients who entered the AKD phase, including patients who died or initiated chronic KRT between day 8 and day 90 in the non-recovery group.

**Additional File 9: Figure S6:** Correlation matrix displaying the strength of association between pairs of candidate risk factors.

**Additional File 10: Table S6:** Association between progression to AKD (versus early recovery) and subsequent risk of death or development of *de novo* CKD within the year following the AKI episode (results of multivariable Cox model).

**Figure S1:** Kaplan-Meier survival curve comparing post-AKI survival according to AKI category (N=56,906). ^[[1]](#footnote-1)^


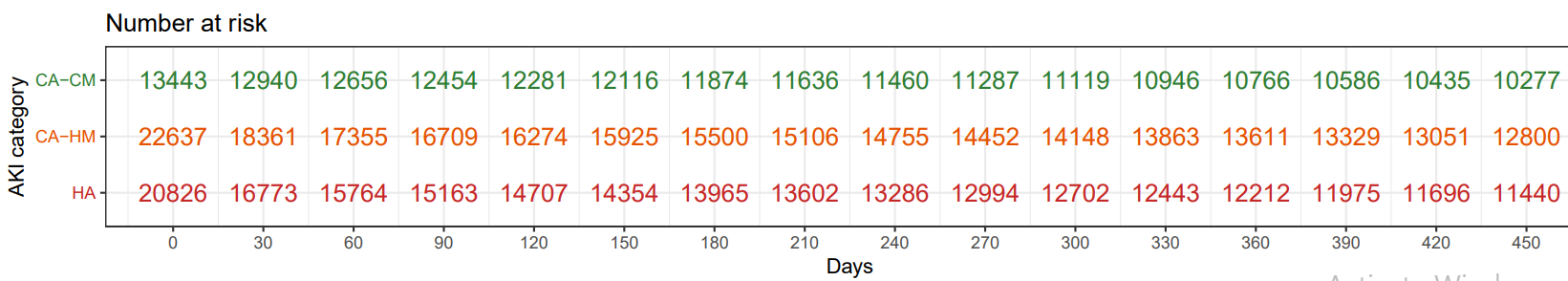


Days

**
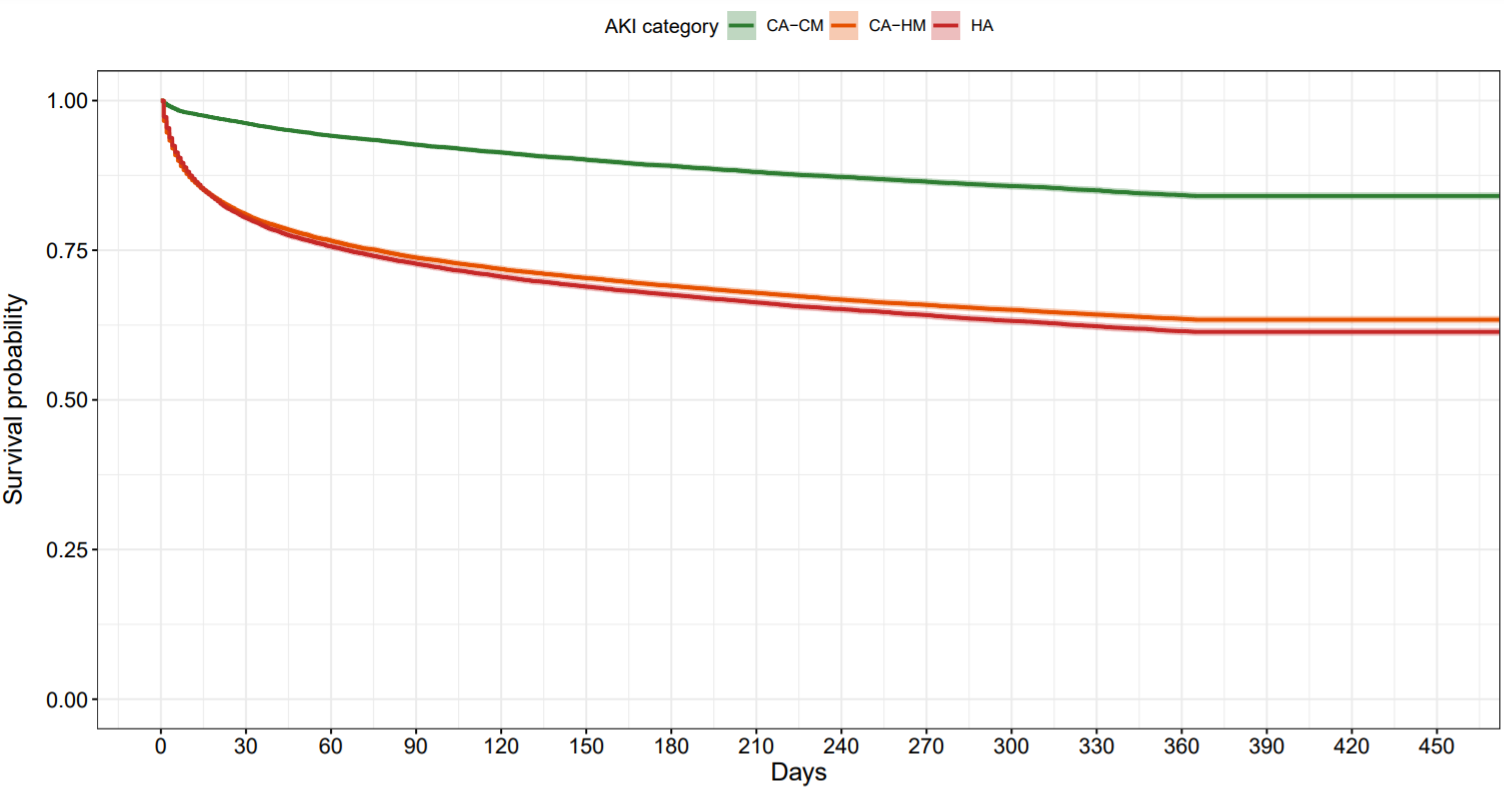
**

**Table S1**: Patients’ recovery status at 7- and 90-days post-AKI (%)

|  | **Creatinine-recovery** | **No creatinine-recovery** | **Death** | **KRT** | **Untested** | **Total** |
| --- | --- | --- | --- | --- | --- | --- |
| **Patients’ status at day 7** | | |  |  |  |  |
| **Community-acquired/community-managed AKI** | | |  |  |  |  |
| Stage 1 | 1,231 (10.3) | 2,276 (19.0) | 145 (1.2) | <5 | 8,332 (69.5) | 11,984 (100) |
| Stage 2 | 140 (12.5) | 425 (38.0) | 63 (5.6) | <5 | 490 (43.8) | 1,118 (100) |
| Stage 3 | 27 (7.9) | 124 (36.4) | 35 (10.3) | <5 | 151 (44.3) | 341 (100) |
| All stages | 1,398 (10.4) | 2,825 (21.0) | 243 (1.8) | <5 | 8,973 (66.7) | 13,443 (100) |
| **Community-acquired/hospital-managed AKI** | | |  |  |  |  |
| Stage 1 | 7,624 (49.5) | 4,605 (29.9) | 1,370 (8.9) | <5 | 1,785 (11.6) | 15,385 (100) |
| Stage 2 | 2,035 (46.1) | 1,589 (36.0) | 640 (14.5) | <5 | 148 (3.3) | 4,412 (100) |
| Stage 3 | 986 (34.7) | 1,333 (46.9) | 475 (16.7) | 18 (0.63) | 28 (0.98) | 2,840 (100) |
| All stages | 10,645 (47.0) | 7,527 (33.2) | 2,485 (11.0) | 19 (0.084) | 1,961 (8.7) | 22,637 (100) |
| **Hospital-acquired AKI** | | |  |  |  |  |
| Stage 1 | 7,085 (39.4) | 7,185 (39.9) | 1,639 (9.1) | <5 | 2,083 (11.6) | 17,992 (100) |
| Stage 2 | 721 (34.8) | 863 (41.7) | 374 (18.1) | <5 | 110 (5.3) | 2,069 (100) |
| Stage 3 | 192 (25.1) | 373 (48.8) | 151 (19.7) | 22 (2.9) | 27 (3.5) | 765 (100) |
| All stages | 7,998 (38.4) | 8,421 (40.4) | 2,164 (10.4) | 23 (0.11) | 2,220 (10.7) | 20,826 (100) |
| **All AKI** | 20,041 (35.2) | 18,773 (33.0) | 4,892 (8.6) | 46 (0.081) | 13,154 (23.1) | 56,906 (100) |
| **Patients’ status at day 90** | | |  |  |  |  |
| **Community-acquired/community-managed AKI** | | |  |  |  |  |
| Stage 1 | 993 (43.6) | 673 (29.6) | 191 (8.4) | <5 | 417 (18.3) | 2,276 (100) |
| Stage 2 | 164 (38.6) | 160 (37.6) | 42 (9.9) | <5 | 59 (13.9) | 425 (100) |
| Stage 3 | 37 (29.8) | 54 (43.5) | 14 (11.3) | 17 (13.7) | <5 | 124 (100) |
| All stages | 1,194 (42.3) | 887 (31.4) | 247 (8.7) | 19 (0.67) | 478 (16.9) | 2,825 (100) |
| **Community-acquired/hospital-managed AKI** | | |  |  |  |  |
| Stage 1 | 1,999 (43.4) | 981 (21.3) | 968 (21.0) | 10 (0.22) | 647 (14.0) | 4,605 (100) |
| Stage 2 | 668 (42.0) | 383 (24.1) | 384 (24.2) | <5 | 150 (9.4) | 1,589 (100) |
| Stage 3 | 582 (43.7) | 363 (27.2) | 290 (21.7) | 56 (4.2) | 42 (3.1) | 1,333 (100) |
| All stages | 3,249 (43.2) | 1,727 (22.9) | 1,642 (21.8) | 70 (0.93) | 839 (11.1) | 7,527 (100) |
| **Hospital-acquired AKI** | | |  |  |  |  |
| Stage 1 | 2,794 (38.9) | 2,084 (29.0) | 1,498 (20.8) | 11 (0.15) | 798 (11.1) | 7,185 (100) |
| Stage 2 | 331 (38.3) | 242 (28.0) | 225 (26.1) | <5 | 65 (7.5) | 863 (100) |
| Stage 3 | 130 (34.8) | 119 (31.9) | 83 (22.2) | 29 (7.8) | 12 (3.2) | 373 (100) |
| All stages | 3,255 (38.6) | 2,445 (29.0) | 1,806 (21.4) | 40 (0.47) | 875 (10.4) | 8,421 (100) |
| **All AKI** | 7,698 (41.0) | 5,059 (26.9) | 3,695^[[2]](#footnote-2)^ (19.7) | 129^a^ (0.69) | 2,192 (11.7) | 18,773^[[3]](#footnote-3)^ (100) |

**Table S2:** Characteristics of patients with community-acquired/community-managed AKI tested versus untested within the first seven days post AKI diagnosis, excluding patients who died or initiated chronic KRT within this time period.

|  | **Tested**  **(n=4,223)** | **Untested**  **(n=8,973)** |  |
| --- | --- | --- | --- |
|  |  |  |  |
| **AKI severity at diagnosis** |  |  |  |
| Stage 1 | 3507 (83) | 8332 (93) |  |
| Stage 2 | 565 (13) | 490 (5) |  |
| Stage 3 | 151 (4) | 151 (2) |  |
|  |  |  |  |
| **Age at AKI diagnosis (median, [IQR])** | 72 [57 – 82] | 68 [50 – 79] |  |
| **Sex = Male** | 4706 (37) | 3049 (34) |  |
| **SIMD quintile** |  |  |  |
| 1 (most deprived) | 753 (18) | 1862 (21) |  |
| 2 | 819 (19) | 1856 (21) |  |
| 3 | 815 (19) | 1801 (20) |  |
| 4 | 1130 (27) | 2093 (23) |  |
| 5 (least deprived) | 706 (17) | 1361 (15) |  |
| **AKI identified by** |  |  |  |
| SCr ratio | 4116 (97) | 8856 (99) |  |
| SCr increment | 107 (3) | 117 (1) |  |
| **Baseline eGFR category** |  |  |  |
| ≥ 90 | 1229 (29) | 4223 (47) |  |
| 60 – 89 | 1494 (35) | 2894 (32) |  |
| 45 – 59 | 730 (17) | 978 (11) |  |
| 30 – 44 | 551 (13) | 630 (7) |  |
| < 30 | 219 (5) | 248 (3) |  |
| **Comorbidity** |  |  |  |
| Chronic kidney disease (CKD) | 1603 (38) | 2346 (26) |  |
| Cancer | 1159 (27) | 2117 (24) |  |
| Coronary arterial disease (CAD) | 865 (20) | 1569 (17) |  |
| Congestive heart failure (CHF) | 440 (10) | 726 (8) |  |
| Diabetes | 1133 (27) | 2266 (26) |  |
| Hypertension | 1361 (32) | 2469 (27) |  |
| **Medication in prior 90 days** |  |  |  |
| ACEi/ARB | 1821 (43) | 3313 (37) |  |
| Loop diuretic | 1129 (27) | 1914 (21) |  |
| Metformin | 464 (11) | 949 (11) |  |
| NSAID | 328 (8) | 846 (9) |  |
| Statin | 1537 (36) | 2933 (33) |  |

**Table S3:** 90-day status of patients untested within the first 7 days post AKI diagnosis.

|  | | **Creatinine-recovery** | | **No creatinine-recovery** | **Death** | **KRT** | **Untested** | **Total** |
| --- | --- | --- | --- | --- | --- | --- | --- | --- |
| **Patients’ status at day 90 for patients untested at day 7** | | | | |  |  |  |  |
| **Community-acquired/community-managed AKI** | | | | |  |  |  |  |
| Stage 1 | 2,656 (31.9) | | 2,185 (26.2) | | 364 (4.4) | <5* | 3,124 (37.5) | 8,332 (100) |
| Stage 2 | 145 (29.6) | | 179 (36.5) | | 44 (9.0) | <5* | 119 (24.3) | 490 (100) |
| Stage 3 | 32 (21.2) | | 56 (37.1) | | 26 (17.2) | 19 (12.6) | 18 (11.9) | 151 (100) |
| All stages | 2,833 (31.6) | | 2,420 (27.0) | | 434 (4.8) | 25 (0.3) | 3,261 (36.3) | 8,973 (100) |
| **Community-acquired/hospital-managed AKI** | | | | |  |  |  |  |
| Stage 1 | 664 (37.2) | | 295 (16.5) | | 145 (8.1) | <5* | 681 (38.1) | 1,785 (100) |
| Stage 2 | 52 (35.1) | | 17 (11.5) | | 21 (14.2) | <5* | 58 (39.2) | 148 (100) |
| Stage 3 | 7 (25.0) | | 5 (17.9) | | 5 (17.9) | <5* | 7 (25.0) | 28 (100) |
| All stages | 723 (36.9) | | 317 (16.2) | | 171 (8.7) | <5* | 746 (38.0) | 1,961 (100) |
| **Hospital-acquired AKI** | | | | |  |  |  |  |
| Stage 1 | 714 (34.3) | | 445 (21.4) | | 210 (10.1) | <5* | 714 (34.3) | 2,083 (100) |
| Stage 2 | 29 (26.4) | | 17 (15.4) | | 24 (21.8) | <5* | 40 (36.4) | 110 (100) |
| Stage 3 | 7 (25.9) | | 4 (14.8) | | 8 (29.6) | <5* | 5 (18.5) | 27 (100) |
| All stages | 750 (33.8) | | 466 (21.0) | | 242 (10.9) | <5* | 759 (34.2) | 2,220 (100) |
| **All AKI** | 4,306 (32.7) | | 3,203 (24.3) | | 847 (6.4) | 32 (0.24) | 4,766 (36.2) | 13,154 (100) |

*Count data below 5 are non-disclosable to protect patients’ confidentiality.

**Figure S2**: Forest plot displaying the results of multivariable logistic regression assessing the association between individual predictors and progression to AKD, excluding patients who died or initiated chronic KRT within the first 7 days.**^[[4]](#footnote-4)^**


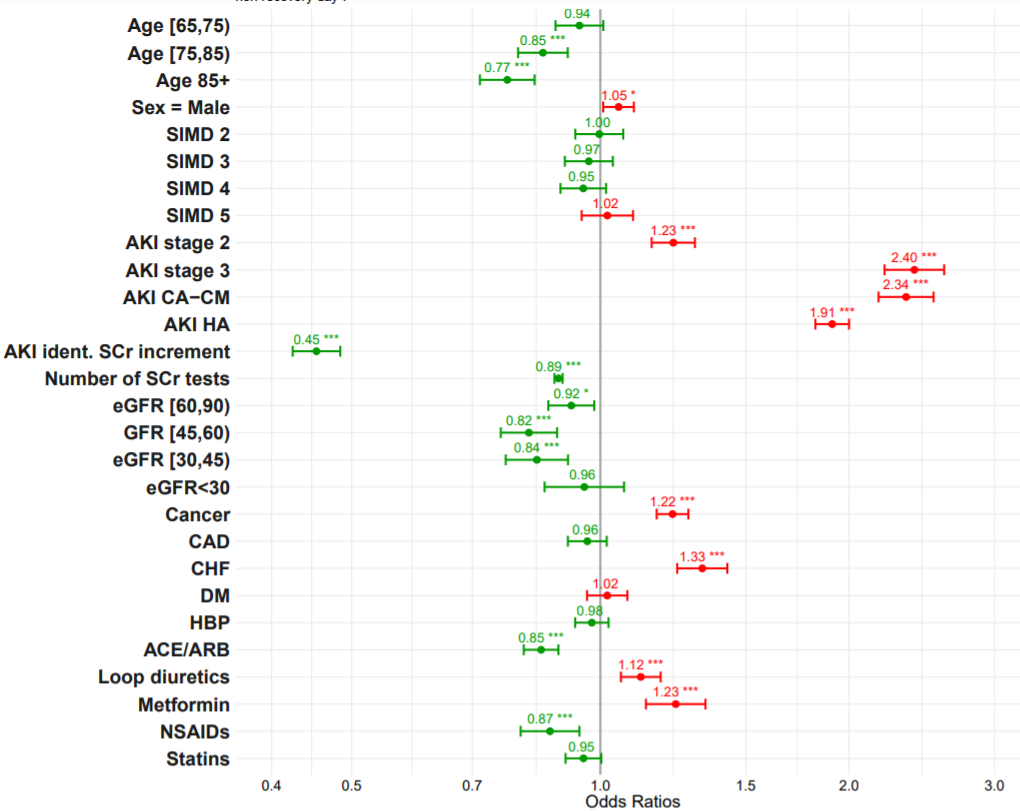


**Table S4**: Results of univariable and multivariable logistic regression assessing the association between individual predictors and non-recovery at day 7, including patients who died or initiated chronic KRT within the first 7 days in the non-recovery group.

|  |  | **Unadjusted** | | **Adjusted** | |
| --- | --- | --- | --- | --- | --- |
|  |  | **OR (95% CI)** | **p-value** | **OR (95% CI)** | **p-value** |
|  | N | 43,752 |  | 43,752 |  |
| **No recovery at day 7, N (%)** |  | 23,711 (54.2%) |  | 23,711 (54.2%) |  |
| **Age at AKI diagnosis** |  |  |  |  |  |
| <65 (Ref) | 11,075 | -- | -- | -- | -- |
| 65-74 | 9,949 | 0.94 (0.89 – 1.00) | 0.04 | 1.00 (0.94 – 1.07) | 0.42 |
| 75-84 | 13,520 | 0.86 (0.82 – 0.91) | <0.001 | 1.00 (0.94 – 1.06) | 0.84 |
| 85+ | 9,208 | 0.86 (0.82 – 0.91) | <0.001 | 0.99 (0.92 – 1.06) | 0.88 |
| **Sex = Male** | 20,995 | 0.98 (0.95 - 1.02) | 0.36 | 1.05 (1.01 – 1.09) | 0.02 |
| **SIMD quintile** |  |  |  |  |  |
| 1 (Ref) | 8,172 | -- | -- | -- | -- |
| 2 | 8,773 | 0.99 (0.93 – 1.05) | 0.64 | 0.99 (0.93 – 1.05) | 0.74 |
| 3 | 8,743 | 0.94 (0.88 – 0.99) | 0.03 | 0.94 (0.88 – 1.00) | 0.04 |
| 4 | 11,201 | 0.91 (0.86 to 0.97) | 0.002 | 0.90 (0.85 – 0.96 | <0.001 |
| 5 | 6,863 | 0.98 (0.92 – 1.04) | 0.51 | 0.97 (0.91 – 1.04) | 0.4 |
| **AKI category** |  |  |  |  |  |
| CA-HM (Ref) | 20,676 | -- | -- | -- | -- |
| CA-CM | 4,470 | 2.33 (2.18 – 2.50) | <0.001 | 2.56 (2.39 – 2.75) | <0.001 |
| HA | 18,606 | 1.41 (1.35 – 1.46) | <0.001 | 1.76 (1.69 – 1.84) | <0.001 |
| **AKI identified by** |  |  |  |  |  |
| SCr ratio (Ref) | 37,410 | -- | -- | -- | -- |
| SCr increment | 6,342 | 0.43 (0.40 - 0.46) | <0.001 | 0.47 (0.44 – 0.49) | <0.001 |
| **AKI severity at diagnosis** |  |  |  |  |  |
| Stage 1 (Ref) | 33,161 | -- | -- | -- | -- |
| Stage 2 | 6,851 | 1.26 (1.20 - 1.33) | <0.001 | 1.27 (1.21 – 1.35) | <0.001 |
| Stage 3 | 3,740 | 1.95 (1.81 - 2.09) | <0.001 | 2.26 (2.10 – 2.44) | <0.001 |
| **Number of tests** |  | 0.81 (0.81 – 0.82) | <0.001 | -- | -- |
| **Baseline eGFR category** |  |  |  |  |  |
| ≥ 90 (Ref) | 9,918 | -- | -- | -- | -- |
| 60 – 89 | 17,175 | 0.84 (0.80 - 0.88) | <0.001 | 0.89 (0.84 – 0.95) | <0.001 |
| 45 – 59 | 7,913 | 0.71 (0.67 - 0.75) | <0.001 | 0.79 (0.74 – 0.85) | <0.001 |
| 30 – 44 | 6,007 | 0.68 (0.64 - 0.72) | <0.001 | 0.79 (0.73 – 0.86) | <0.001 |
| < 30 | 2,739 | 0.79 (0.73 – 0.86) | <0.001 | 0.90 (0.82 – 1.00) | 0.05 |
| **Comorbidity** |  |  |  |  |  |
| Cancer | 15,778 | 1.25 (1.21 - 1.30) | <0.001 | 1.29 (1.24 – 1.34) | <0.001 |
| CAD | 11,271 | 0.96 (0.92 - 1.00) | 0.05 | 1.01 (0.96 – 1.06) | 0.74 |
| CHF | 5,685 | 1.22 (1.15 - 1.29) | <0.001 | 1.34 (1.25 – 1.43) | <0.001 |
| Diabetes | 11,992 | 0.97 (0.93 - 1.01) | 0.12 | 1.00 (0.94 – 1.05) | 0.89 |
| Hypertension | 16,843 | 0.91 (0.88 – 0.95) | <0.001 | 0.97 (0.93 – 1.02) | 0.23 |
| **Medication in prior 90 days** |  |  |  |  |  |
| ACEi/ARB | 16,190 | 0.78 (0.75 - 0.81) | <0.001 | 0.78 (0.75 – 0.82) | <0.001 |
| Loop diuretic | 9,973 | 1.08 (1.04 - 1.13) | <0.001 | 1.16 (1.10 – 1.22) | <0.001 |
| Metformin | 4,232 | 1.04 (0.98 - 1.11) | 0.19 | 1.17 (1.08 – 1.27) | <0.001 |
| NSAID | 2,981 | 0.91 (0.85 - 0.97) | 0.016 | 0.87 (0.80 – 0.94) | <0.001 |
| Statin | 15,931 | 0.85 (0.82 - 0.89) | <0.001 | 0.93 (0.89 – 0.98) | 0.003 |

**Figure S3**: Forest plot displaying the results of multivariable logistic regression assessing the association between individual predictors and non-recovery at day 7, including patients who died or initiated chronic KRT within the first 7 days in the non-recovery group. **^[[5]](#footnote-5)^**

**
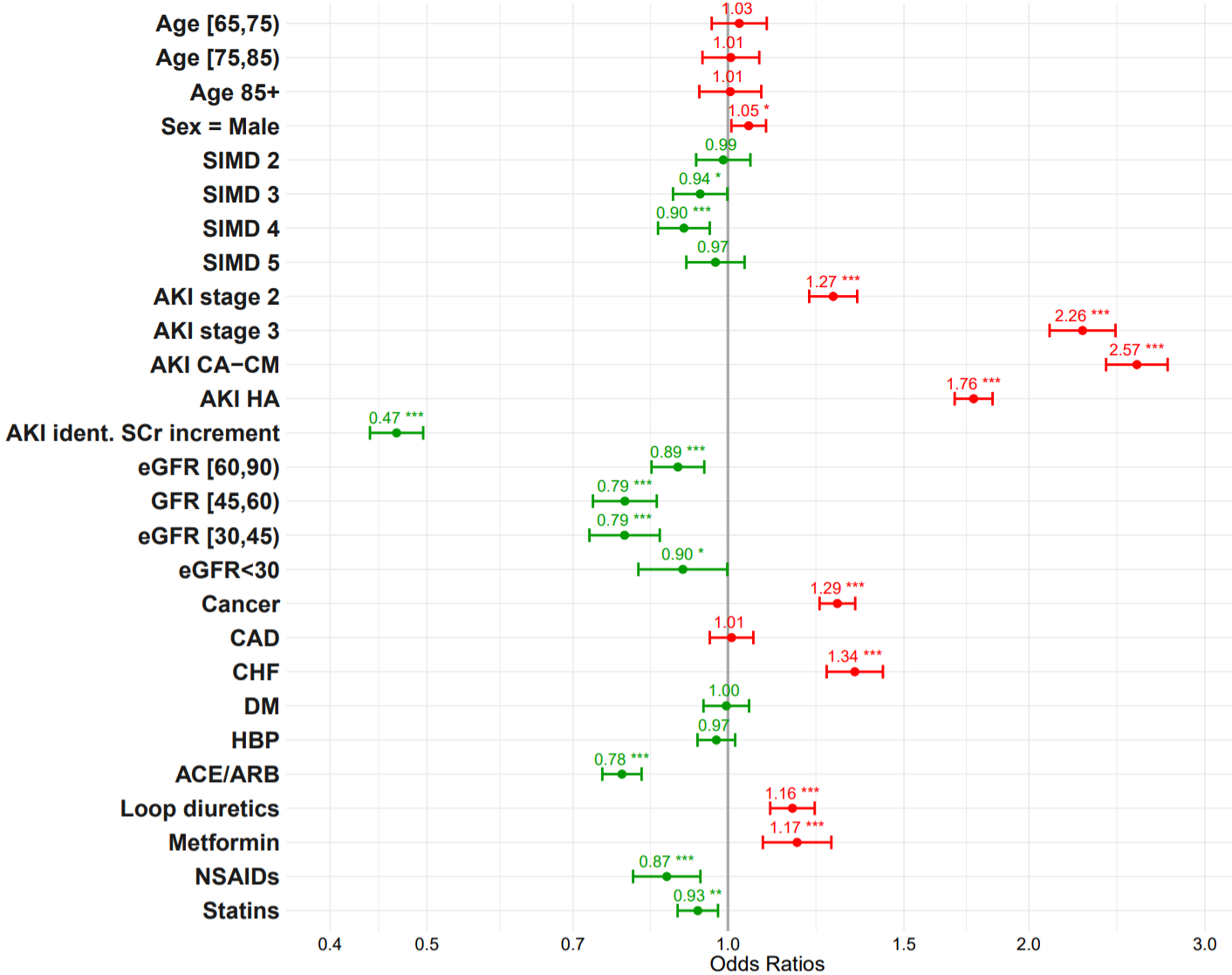
**

**Figure S4**: Forest plot displaying the results of multivariable logistic regression assessing the association between individual predictors and non-recovery at day 90 in patients who entered the AKD phase, excluding patients who died or initiated chronic KRT between day 8 and day 90. ^[[6]](#footnote-6)^


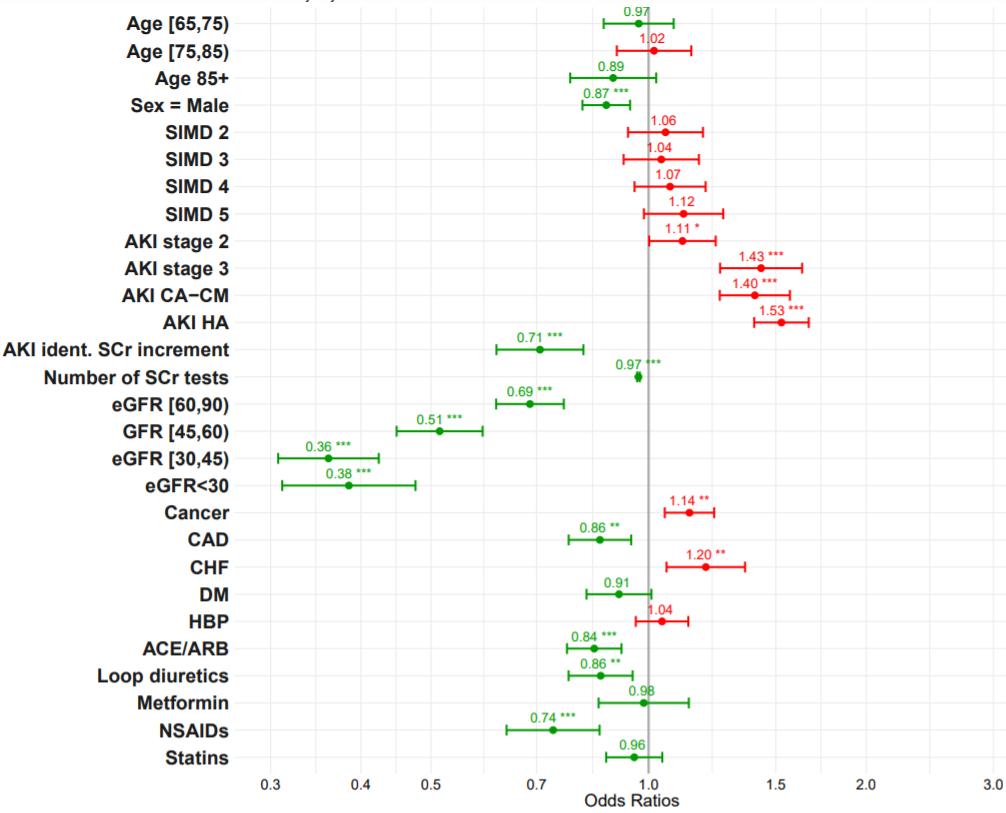


**Table S5**: Results of univariable and multivariable logistic regression assessing the association between individual predictors and non-recovery at day 90 in patients who entered the AKD phase, including patients who died or initiated chronic KRT between day 8 and day 90 in the non-recovery group.

|  |  | **Unadjusted** | | **Adjusted** | |
| --- | --- | --- | --- | --- | --- |
|  |  | **OR (95% CI)** | **p-value** | **OR (95% CI)** | **p-value** |
|  | N | 16,581 |  | 16,581 |  |
| **No recovery at day 90, N (%)** |  | 8,883 (53.6%) |  | 8,883 (53.6%) |  |
| **Age at AKI diagnosis** |  |  |  |  |  |
| <65 (Ref) | 4,161 | -- | -- | -- | -- |
| 65-74 | 3,832 | 0.95 (0.87 – 1.04) | 0.2 | 1.17 (1.06-1.29) | 0.002 |
| 75-84 | 4,986 | 0.94 (0.87 – 1.02) | 0.1 | 1.30 (1.17-1.44) | <0.001 |
| 85+ | 3,602 | 1.09 (1.00 – 1.20) | 0.05 | 1.48 (1.32-1.66) | <0.001 |
| **Sex = Male** | 7,975 | 0.89 (0.83 – 0.94) | <0.001 | 0.91 (0.86-0.97) | 0.005 |
| **SIMD quintile** |  |  |  |  |  |
| 1 (Ref) | 3,094 | -- | -- | -- | -- |
| 2 | 3,312 | 1.05 (0.95 – 1.16) | 0.3 | 1.05 (0.95-1.16) | 0.3 |
| 3 | 3,259 | 1.01 (0.91 – 1.11) | 0.8 | 1.00 (0.91-1.11) | 0.9 |
| 4 | 4,233 | 1.01 (0.92 – 1.11) | 0.8 | 0.98 (0.89-1.08) | 0.7 |
| 5 | 2,683 | 1.06 (0.96 – 1.18) | 0.3 | 1.01 (0.91-1.13) | 0.8 |
| **AKI category** |  |  |  |  |  |
| CA-HM (Ref) | 6,688 | -- | -- | -- | -- |
| CA-CM | 2,347 | 0.91 (0.83 – 1.00) | 0.06 | 0.99 (0.90-1.09) | 0.8 |
| HA | 7,546 | 1.24 (1.17 – 1.33) | <0.001 | 1.28 (1.19-1.37) | <0.001 |
| **AKI identified by** |  |  |  |  |  |
| SCr ratio (Ref) | 14,880 | -- | -- | -- | -- |
| SCr increment | 1,701 | 0.69 (0.63 - 0.77) | <0.001 | 0.71 (0.63-0.79) | <0.001 |
| **AKI severity at diagnosis** |  |  |  |  |  |
| Stage 1 (Ref) | 12,204 | -- | -- | -- | -- |
| Stage 2 | 2,603 | 1.12 (1.02 - 1.21) | 0.01 | 1.17 (1.07-1.28) | <0.001 |
| Stage 3 | 1,774 | 1.23 (1.11 – 1.36) | <0.001 | 1.42 (1.28-1.59) | <0.001 |
| **Number of tests** | Median = 5 [IQR: 2 – 11] | 0.97 (0.97 – 0.98) | <0.001 | -- | -- |
| **Baseline eGFR category** |  |  |  |  |  |
| ≥ 90 (Ref) | 3,923 | -- | -- | -- | -- |
| 60 – 89 | 6,621 | 0.79 (0.73 - 0.86) | <0.001 | 0.71 (0.65-0.78) | 0.01 |
| 45 – 59 | 2,851 | 0.65 (0.59 - 0.72) | <0.001 | 0.58 (0.52-0.65) | <0.001 |
| 30 – 44 | 2,144 | 0.57 (0.51 - 0.63) | <0.001 | 0.50 (0.44-0.57) | <0.001 |
| < 30 | 1,042 | 0.76 (0.66 – 0.87) | <0.001 | 0.64 (0.55-0.76) | 0.4 |
| **Comorbidity** |  |  |  |  |  |
| Cancer | 6,311 | 1.44 (1.35 - 1.53) | <0.001 | 1.42 (1.33-1.52) | <0.001 |
| CAD | 4,211 | 0.89 (0.83 – 0.95) | <0.001 | 0.96 (0.88-1.04) | 0.3 |
| CHF | 2,361 | 1.09 (1.00 - 1.19) | 0.06 | 1.27 (1.15-1.41) | <0.001 |
| Diabetes | 4,789 | 0.79 (0.74 – 0.85) | <0.001 | 0.99 (0.91-1.08) | 0.8 |
| Hypertension | 6,376 | 0.91 (0.85 – 0.97) | 0.003 | 1.01 (0.95-1.09) | 0.7 |
| **Medication in prior 90 days** |  |  |  |  |  |
| ACEi/ARB | 6,201 | 0.65 (0.61 - 0.70) | <0.001 | 0.76 (0.70-0.81) | <0.001 |
| Loop diuretic | 3,968 | 0.94 (0.87 - 1.00) | 0.07 | 1.08 (0.99-1.17) | 0.07 |
| Metformin | 1,827 | 0.68 (0.62 – 0.75) | <0.001 | 0.85 (0.75-0.96) | 0.009 |
| NSAID | 1,110 | 0.85 (0.75 – 0.96) | 0.008 | 0.83 (0.73-0.94) | 0.003 |
| Statin | 6,033 | 0.72 (0.68 - 0.77) | <0.001 | 0.88 (0.81-0.94) | <0.001 |

**Figure S5**: Forest plot displaying the results of multivariable logistic regression assessing the association between individual predictors and non-recovery at day 90 in patients who entered the AKD phase, including patients who died or initiated chronic KRT between day 8 and day 90 in the non-recovery group. ^[[7]](#footnote-7)^


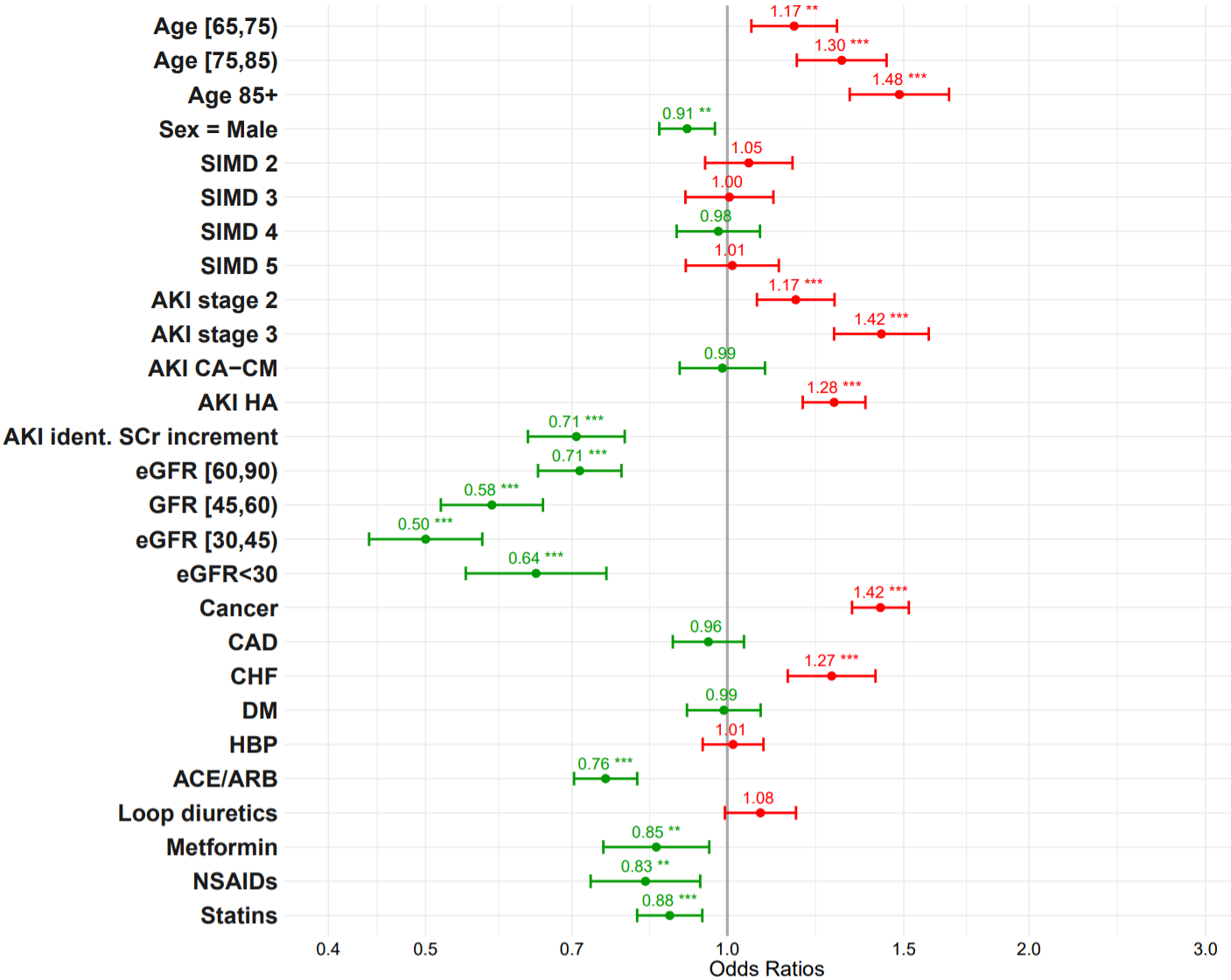


**Figure S6:** Correlation matrix displaying the strength of association between pairs of candidate risk factors.

**
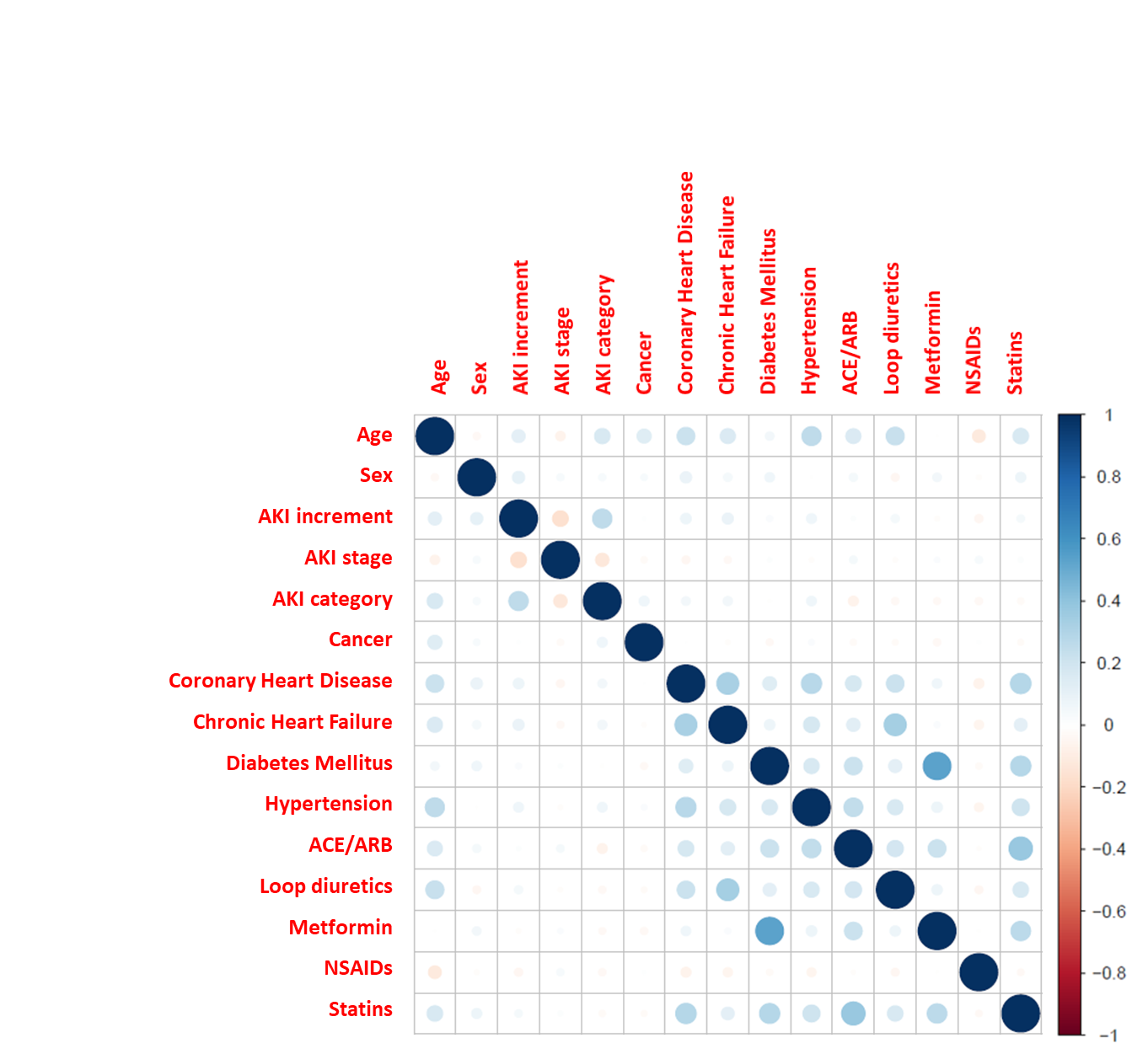
**

**Table S6:** Association between progression to AKD (versus early recovery) and subsequent risk of death or development of *de novo* CKD within the year following the AKI episode (results of multivariable Cox model).

|  | **N** | **HR for 1-year mortality*** | **N** | **HR for 1-year risk of de novo CKD*** |
| --- | --- | --- | --- | --- |
| **All** | 29,330 | 1.20 (1.13 to 1.26) | 14,486 | 2.21 (1.91 to 2.57) |
| **CA-CM** | 2,682 | 1.55 (1.23 to 1.95) | 1,334 | 3.25 (1.99 to 5.31) |
| **CA-HM** | 14,624 | 1.28 (1.18 to 1.39) | 7,890 | 2.12 (1.72 to 2.60) |
| **HA** | 12,024 | 1.14 (1.06 to 1.24) | 5,262 | 2.00 (1.57 to 2.55) |

*Adjusted for all baseline covariates (age, sex, SIMD, baseline eGFR, comorbidities and previous medications), frequency of SCr tests, AKI stage and recovery eGFR category (+AKI category when pooling all categories together).

1. *CA-CM: Community-acquired/community-managed AKI; CA-HM: Community-acquired/hospital-managed AKI; HA: Hospital-acquired AKI* [↑](#footnote-ref-1)
2. 6 patients initiated KRT and then died during the 8 to 90 days period, their status was considered as “Death” [↑](#footnote-ref-2)
3. Patients who entered AKD phase, i.e: have been tested at day 7 but did not recover within 7 days, did not die or initiate RRT during first 7 days.

   * Count data below 5 are non-disclosable in order to protect patient’s confidentiality. [↑](#footnote-ref-3)
4. *Variables associated with progression to AKD are in red whilst those associated with early recovery are in green. Significance level: * <0.05, **<0.01, *** <0.001* [↑](#footnote-ref-4)
5. *Variables associated with non-recovery at day 7 are in red whilst those associated with early recovery (between day 1 and day 7) are in green. Significance level: * <0.05, **<0.01, *** <0.001.* [↑](#footnote-ref-5)
6. *Variables associated with non-recovery at day 90 are in red whilst those associated with recovery during the AKD phase (between day 8 and day 90) are in green. Significance level: * <0.05, **<0.01, *** <0.001.* [↑](#footnote-ref-6)
7. *Variables associated with non-recovery at day 90 are in red whilst those associated with recovery during the AKD phase (between day 8 and day 90) are in green. Significance level: * <0.05, **<0.01, *** <0.001.* [↑](#footnote-ref-7)
